# New insights into the analgesic properties of the XCL1/XCR1 and XCL1/ITGA9 axes modulation under neuropathic pain conditions - evidence from animal studies

**DOI:** 10.3389/fimmu.2022.1058204

**Published:** 2022-12-22

**Authors:** Agata Ciechanowska, Ewelina Rojewska, Anna Piotrowska, Justyna Barut, Katarzyna Pawlik, Katarzyna Ciapała, Grzegorz Kreiner, Joanna Mika

**Affiliations:** ^1^ Department of Pain Pharmacology, Maj Institute of Pharmacology, Polish Academy of Sciences, Kraków, Poland; ^2^ Department of Brain Biochemistry, Maj Institute of Pharmacology, Polish Academy of Sciences, Kraków, Poland

**Keywords:** XCL1, XCR1, ITGA9, CCI, astroglia, chemokine, opioid, microglia

## Abstract

Recent studies have indicated the involvement of chemokine-C-motif ligand 1 (XCL1) in nociceptive transmission; however, the participation of its two receptors, canonical chemokine-C-motif receptor 1 (XCR1) and integrin alpha-9 (ITGA9), recently recognized as a second receptor, has not been clarified to date. The aim was to explore by which of these receptors XCL1 reveals its pronociceptive properties and how the XCL1-XCR1 and XCL1-ITGA9 axes blockade/neutralization influence on pain-related behavior and opioid analgesia in the model of neuropathic pain. In our studies we used Albino Swiss mice which were exposed to the unilateral sciatic nerve chronic constriction injury (CCI) as a neuropathic pain model. Animals received single intrathecal (*i.t.*) injection of XCL1, XCL1 neutralizing antibodies, antagonist of XCR1 (vMIP-II) and neutralizing antibodies of ITGA9 (YA4), using lumbar puncture technique. Additionally we performed *i.t.* co-administration of abovementioned neutralizing antibodies and antagonists with single dose of morphine/buprenorphine. To assess pain-related behavior the von Frey and cold plate tests were used. To measure mRNA and protein level the RT-qPCR and Western Blot/Elisa/immunofluorescence techniques were performed, respectively. Statistical analysis was conducted using ANOVA with a Bonferroni correction. Presented studies have shown time-dependent upregulation of the mRNA and/or protein expression of XCL1 in the spinal cord after nerve injury as measured on day 1, 4, 7, 14, and 35. Our immunofluorescence study showed that XCL1 is released by astroglial cells located in the spinal cord, despite the neural localization of its receptors. Our results also provided the first evidence that the blockade/neutralization of both receptors, XCR1 and ITGA9, reversed hypersensitivity after intrathecal XCL1 administration in naive mice; however, neutralization of ITGA9 was more effective. In addition, the results proved that the XCL1 neutralizing antibody and, similarly, the blockade of XCR1 and neutralization of ITGA9 diminished thermal and mechanical hypersensitivity in nerve injury-exposed mice after 7 days. Additionally, neutralization of XCL1 improves morphine analgesia. Moreover, blockade of XCR1 positively influences buprenorphine effectiveness, and neutralization of ITGA9 enhances not only buprenorphine but also morphine analgesia. Therefore, blockade of the XCL1-ITGA9 interaction may serve as an innovative strategy for the polypharmacotherapy of neuropathic pain in combination with opioids.

## 1 Introduction

Neuropathic pain affects 10% of the world’s population (1) and is caused by many factors, including mechanical injury to the peripheral or central nervous system. The related complaints require better understanding, diagnosis and treatment because the current therapy is unsatisfactory. Unfortunately, patients with neuropathic pain are less sensitive to opioid drugs, which are the most powerful painkillers currently available in clinics (2). This makes the development of new strategies for pharmacotherapy toward painful neuropathies an urgent need. Such progress requires an extensive understanding of the molecular and cellular mechanisms involved in the development of chronic pain originating from peripheral nerve injury (3). Glia have an undeniable role in the maintenance of homeostasis in the nervous system. Depending on the nature of the stimulus, glial cells can take on a number of activation states, which consequently causes altered gene expression and changes in morphology and function (4). Microglia represent resident immune cells of the central nervous system (CNS), revealing a classically activated phenotype associated with the release of proinflammatory molecules after chronic activation, contributing to neurodegeneration (5). Additionally, astroglia are a population of cells that play an integral role in maintaining CNS homeostasis. Their activation may result in the development of neurodegenerative disorders and is important in the modulation of neuropathic pain (6). This is why the pharmacological modulation of the abovementioned interactions is very effective in relieving painful symptoms in a neuropathic pain model (7). Minocycline (MC), which is one of the most potent substances causing inhibitory effects on the release of pronociceptive factors by glia (8), has the potential to treat the symptoms of neuropathic pain of different etiologies, e.g., in animal models such as streptozotocin (STZ)-induced diabetes (7) and chronic constriction injury (CCI) of the sciatic nerve (9–12). MC was also shown to influence important pain-related intracellular pathways, especially what was well studied, it beneficially influences p38 mitogen-activated protein kinases (MAPK) in an animal model of inflammatory and neuropathic pain (13, 14). Moreover, MC suppresses the increased gene expression of CXCL13, CXCL1, CCL2, CXCL11, and CCL7 after CCI (15). The release of chemokines by neuronal and nonneuronal cells such as microglia/astroglia is an important factor underlying neuroimmune crosstalk during neuropathic pain development and maintenance (4, 16, 17). Our previous studies showed that the neutralization of some chemokines [e.g., CCL1 (18), CCL2 (19), CCL3 (20), CCL7 (19), and CCL9 (20)] and blockade of several receptors [e.g., CCR1 (21), CCR2 (22), CCR3 (23), CCR4 (24), CCR5 (25), CXCR2 and CXCR3 (26)] in animal models of neuropathic pain diminish the development of symptoms; however, the role of XCL1 and its receptors is still unknown.

In our previous study, we demonstrated the spatiotemporal upregulation of XCL1 in several areas of the murine brain (cortex, thalamus, and hippocampus), which began shortly after traumatic brain injury model induction and persisted until up to 5 weeks in the cortex (27). This finding indicates that this chemokine may play a key role in neurodegenerative processes. In 2016, we showed for the first time the important role of XCL1 in diabetic neuropathy (7). To date, it was known that XCL1 is released by some immune cells (28) and it was shown that there is an elevated level of XCL1 protein in primary murine astroglial cells after LPS treatment (27). XCL1 acts through a G-protein coupled receptor, XCR1 (29). For a long time, XCR1 was the only known receptor for XCL1. Recently, Matsumoto et al. showed that XCL1 affects fibroblast migration through the heterodimeric (αβ) transmembrane receptor ITGA9 (30), which opened new research horizons in this field. ITGA9 was proposed as a therapeutic target in autoimmune diseases (31). It remains unknown how XCR1 and ITGA9 are involved in nociceptive transmission; however, their role seems to be extremely important in neuropathy, as our previous research proved the strong pronociceptive properties of their ligand, XCL1, in naive animals (7).

We hypothesized that XCL1 may be significant in neuropathic pain development, acting both through XCR1 and ITGA9. For this purpose, we measured spinal mRNA/protein time-course changes in XCL1, XCR1 and ITGA9 in mice after CCI. Moreover, we performed behavioral tests to evaluate the influence of XCL1 neutralizing antibody (nAb) on mechanical and thermal hypersensitivity and morphine analgesia in CCI-exposed mice. Additionally, we determined the impact of consecutive MC treatment (twice daily, 7 days) on hypersensitivity and the levels of IBA1, GFAP, XCL1, XCR1 and ITGA9 after CCI. An additional aim of this study was to determine whether and how XCR1 and ITGA9 blockade/neutralization influence thermal and mechanical hypersensitivity evoked by intrathecally administered XCL1. We also examined the abovementioned receptor blockade/neutralization effects on hypersensitivity development and opioid analgesia 7 days after CCI. To explain the source of XCL1 and the localization of its receptors in the spinal cord, we performed thorough immunofluorescence studies to determine the cellular localization of XCL1/XCR1/ITGA9 in the spinal cord 7 days after CCI.

## 2 Materials and methods

### 2.1 Animals

For our experiments, we used male Albino Swiss adult mice (Charles River, Göttingen, Germany; 9–11 weeks old, weighing 20–25 g). The housing conditions were as follows: 6-10 mice per cage; free access to food and water; temperature of 22 ± 2°C; relative humidity 55 ± 10%; 12-h light/dark cycle. All performed procedures were accomplished with the recommendations of the International Association for the Study of Pain (IASP) and the National Institutes of Health (NIH) Guide for the Care and Use of Laboratory Animals and were approved by the Ethical Committee of the Maj Institute of Pharmacology of the Polish Academy of Sciences (permission numbers: 75/2017, 305/2017, 235/2020, 236/2021, 297/2021, 89/2021, 98/2022). The number of animals was reduced to the essential minimum according to the 3R policy.

### 2.2 Chronic constriction injury

We performed chronic constriction injury (CCI) of the sciatic nerve as a neuropathic pain model, in accordance with Bennett and Xie (1988) (32), modified by Mika et al. (2007) (33). The animals were anesthetized by inhalation of isoflurane (induction, 3%; maintenance, 3%). In brief, there was an incision made below the right hip bone, parallel to the sciatic nerve. The exposed sciatic nerve was loosely tied around the nerve with three ligatures (4/0 silk). The strength of the first knot was dictated by the occurrence of a short contraction in the corresponding hind limb, and the subsequent contractions were performed similarly. All mice developed neuropathic pain-related behaviors (tactile and thermal hypersensitivity).

### 2.3 Pharmacological studies

#### 2.3.1 Intrathecal and intraperitoneal drug administrations

The intrathecal (*i.t.*) injection was performed according to the method described by Hylden and Wilcox (34) and is a standard procedure in our laboratory (18, 26). A Hamilton syringe with a thin needle (0.3 x 13 mm) was used for administration. The substances used in the experiments were injected in a volume of 5 μl between the L5 and L6 vertebrae (the lumbar region of the spinal cord) until symptoms of correct administration (the tail reflex) were observed. The intraperitoneal (*i.p.)* administered substances were injected with a needle size of 0.45 x 12 mm in terms of body weight and were supposed to be located in the peritoneal cavity.

#### 2.3.2 Single intrathecal administration of an XCL1 neutralizing antibody in mice with chronic constriction injury-induced neuropathy

A single *i.t.* administrations of XCL1 nAb (Mouse XCL1/Lymphotactin Antibody; AF486, R&D Systems; Minneapolis, United States) at doses of 1, 4, 8 and 16 μg/5 μl were performed 7 days after CCI, when mechanical and thermal hypersensitivity had been fully developed. The effect of XCL1 nAb administration on the development of tactile hypersensitivity was measured using the von Frey test, while thermal hypersensitivity was measured using the cold plate test after 1, 4, 24, 48 and 96 hours. XCL1 nAb was dissolved in PBS (Merck; Darmstadt, Germany), and PBS was used as a vehicle (V).

#### 2.3.3 Single intrathecal administration of an XCL1 neutralizing antibody with morphine or buprenorphine in mice with chronic constriction injury-induced neuropathy

The *i.t.* administration of XCL1 nAb (8 μg/5 μl) followed by *i.t.* administration of morphine (M, TEVA; Krakow, Poland) or buprenorphine (B, Polfa S. A; Warsaw, Poland) (2.5 μg/5 μl) was performed 7 days after CCI, when we observed the highest level of XCL1 in the spinal cord and fully developed mechanical and thermal hypersensitivity. XCL1 nAb was administered once, at the dose set up based on previously obtained results. The doses of opioids used for the experiment were set up based on our previous studies (23). First, groups of tested animals received *i.t.* administration of vehicle (PBS) or XCL1 nAb. Next, 2 h after V or XCL1 nAb administration, there was a second *i.t.* injection of vehicle (W, water for injections), M or B. Von Frey and cold plate tests were performed 0.5 hours after the second administration (of W, M or B) and 2.5 hours after the first administration (of PBS or XCL1 nAb).

#### 2.3.4 Chronic intraperitoneal administration of minocycline in mice with chronic constriction injury-induced neuropathy

Minocycline hydrochloride (MC; Merck) was dissolved in water for injections (W); therefore, the control mice received W according to the same schedule. The MC was first preemptively administered 16 h and 1 h *i.p*. before CCI surgery and then twice daily for 7 days at a dose of 30 mg/kg. The behavioral tests were conducted 30 min after the last MC administration and 7 days after CCI.

#### 2.3.5 Single intrathecal administration of YA4 or vMIP-II preceded by pronociceptive *i.t.* injection of XCL1 in naive mice

Recombinant mouse chemokine-C-motif ligand 1/lymphotactin protein (XCL1; R&D Systems), recombinant Viral MIP-II protein (vMIP-II, XCR1 antagonist; R&D Systems) and anti-integrin α9 monoclonal antibody (YA4; Fujifilm, Tokyo, Japan) were dissolved in PBS. First, groups of tested animals received *i.t.* administration of vehicle (V; PBS) or XCL1 (X) at a dose of 100 ng/5 μl, which is known to be pronociceptive (7). Next, 2 h after V or XCL1 administration, there was a second *i.t*. administration of V, vMIP-II or YA4 (0.05, 0.5, 1 μg/5 μl). Von Frey and cold plate tests were performed 1, 4, 24, 96 hours after the second administration (V, vMIP-II or YA4), which represents 3, 6, 26 and 98 hours after the first administration (V or XCL1).

#### 2.3.6 Single intrathecal administration of YA4 and vMIP-II in mice with chronic constriction injury-induced neuropathy

vMIP-II and YA4 were dissolved in PBS and administered to mice 7 days after CCI, while the control group received PBS. A single dose of vMIP-II (1 μg/5 μl) or YA4 (1 μg/5 μl), established during the aforementioned experiment, was administered, and behavioral tests were performed after 1, 4, 24, and 96 hours.

#### 2.3.7 Single intrathecal administration of YA4 or vMIP-II with morphine or buprenorphine in mice with chronic constriction injury-induced neuropathy

The experiment aimed to establish the influence of vMIP-II and YA4 on morphine and buprenorphine analgesia 7 days after CCI. First, groups of tested animals received *i.t.* administration of vehicle (V; PBS), vMIP-II or YA4 (1 μg/5 μl, respectively). Next, 3 h after V, vMIP-II or YA4 administration, there was a second *i.t*. administration of vehicle (W; water for injections), M or B (2.5 μg/5 μl). Von Frey and cold plate tests were performed 0.5 hours after the second administration (of W, M or B) and 3.5 hours after the first administration (of V, vMIP-II or YA4).

### 2.4 Behavioral tests

#### 2.4.1 von frey test

Tactile hypersensitivity was measured using calibrated nylon monofilaments (ranging from 0.6 to 6 g; Stoelting, Wood Dale, USA) to observe reactions to mechanical stimuli as previously described (33). The mice were placed in plastic cages with a wire mesh floor before the experiment. After 5 min of adaptation, von Frey filaments were used in order of increasing pressure [g], and they were applied to the midplantar surface of the ipsilateral (right) hind paw (or both hind paws in case of naive mice) until it was lifted. Control mice were tested in the same way.

#### 2.4.2 Cold plate test

Thermal hypersensitivity was measured using a cold plate/hot plate analgesia meter (Ugo Basile; Gemonio, Italy). The temperature of the plate surface was kept at 2°C, and the maximal time (cutoff) possible for the mouse to be kept on the plate surface was 30 seconds. The animals were placed on a cold plate until the (right) hind paw (or both hind paws in case of naive mice) was lifted as previously described (33). The latency was recorded, and the animals were immediately removed from the plate. In every animal exposed to CCI, the injured foot was the first one to react. Control mice were tested in the same way.

### 2.5 Biochemical tests

#### 2.5.1 Analysis of gene expression by RT−qPCR

The lumbar (L4–L6) region of the spinal cord was removed from CCI- and naive mice (sacrificed at 1, 4, 7, 14, 35 days). After decapitation, the tissue was dissected, placed into 1.5 ml plastic Eppendorf tubes with RNAlater (Invitrogen; Waltham, USA), frozen and stored at −80°C. For the synthesis of cDNA, 1000 ng of total RNA was reverse transcribed in a total reaction volume of 20 μl with oligo(dT) primer (Fermentas; Warsaw, Poland) using an Omniscript RT Kit (Qiagen; Hilden, Germany). The cDNA was diluted 1:10 with H_2_O. For each reaction, 50 ng of cDNA was synthesized from the total RNA template of each individual animal and used for quantitative real-time PCR (RT−qPCR). RT−qPCR was run on a Real-Time PCR iCycler (Bio-Rad; Hercules, USA) using Assay-On-Demand TaqMan probes (Thermo Fisher Scientific; Waltham, USA). The amplification efficiency for each assay was determined by running a standard dilution curve. The following TaqMan primer was used: Mm00434772_m1 (*Xcl1*). The expression of the hypoxanthine guanine phosphoribosyl transferase 1 (Hprt1, Mm00446968_m1) transcript was quantified to control for variation in cDNA amounts. The cycle threshold values were automatically calculated using iCycler IQ 3.0 software with the default parameters. The abundance of RNA was calculated as 2^−(threshold cycle)^.

#### 2.5.2 Western blot analysis

The lumbar (L4–L6) regions of the spinal cord were removed from CCI- and naive mice (sacrificed at 1, 7, 35 days) and used for the study. Selected time points represent different phases in injury development – very early, developed and fully established, basing on mRNA analysis. The tissues were placed into 2 ml plastic Eppendorf tubes with RIPA buffer with a protease inhibitor cocktail (inhibitors with broad specificity for various proteases; Merck) and homogenized. Then, the samples were centrifuged (14.000 rpm) for 30 min at 4°C (in the case of time course studies). In the case of the tissue collected from animals chronically treated with minocycline, the lumbar (L4–L6) regions of the spinal cord were placed in tubes with RIPA buffer with protease inhibitor cocktail, homogenized and fractionated in accordance with available protocols, slightly modified by us (35–37). Firstly, the nuclear fraction was separated by centrifugation (2750 rpm) for 5 min at 4°C. The obtained supernatant was then re-centrifuged (8900 rpm) for 5 min at 4°C, after that the pellet contained mitochondria. The supernatant was then centrifuged once again in an ultracentrifuge (28.700 rpm) for 60 min at 4°C resulting in the separation of the membrane (pellet) and cytosolic (supernatant) fractions, which were used for further analyses. The study conducted on two fractions of protein homogenates aimed to differentiate the presence of receptors inside the membrane, which may be changed by the possible internalization - a rapid decrease in the number of cell-surface binding sites in activated cells. The bicinchoninic acid (BCA) method was used to measure the total protein concentration. The samples of protein (10 μg) were then heated for 8 min at 98°C with the addition of loading buffer (4 × Laemmli Buffer; Bio-Rad). Then, the samples were loaded in 4–15% Criterion TGX precast polyacrylamide gels (Bio-Rad) and transferred to Immune-Blot PVDF membranes (Bio-Rad) with the semidry transfer system (30 min, 25 V). Then, the membranes were blocked (5% bovine serum albumin; Merck) in TBST (Tris-buffered saline with 0.1% Tween 20) for 1 h, washed with TBST (4 × 5 min), and incubated overnight with the following commercially available primary antibodies: rabbit anti-XCR1 (1:5000, Lifespan Biosciences; Seattle, USA), rabbit anti-ITGA9 (1:3000, Abcam; Cambridge, Great Britain), mouse anti-β-actin (1:1000; Merck), rabbit anti-IBA1 (1:500, Novus Biologicals; Centennial, USA), and rabbit anti-GFAP (1:10000, Novus Biologicals) at 4°C. Then, the membranes were incubated in anti-rabbit or anti-mouse secondary antibodies (Vector Laboratories; Burlingame, USA) conjugated with horseradish peroxidase at dilutions of 1:5000 for 1 h at room temperature. The primary and secondary antibodies were dissolved in a SignalBoost Immunoreaction Enhancer Kit (Merck). Then, the membranes were washed in TBST (again 4 × 5 min). The detection of immune complexes was attained by the Clarity Western ECL Substrate (Bio-Rad) and visualized with the Fujifilm LAS-4000 Fluor Imager system. The immunoreactive bands obtained in Western blot analysis were quantified using Fujifilm Multi Gauge software.

#### 2.5.3 Enzyme-linked immunosorbent assay analysis

The lumbar (L4–L6) regions of the spinal cord were removed from naive and CCI-exposed mice (sacrificed at 1, 7, 35 days) and used for Enzyme-Linked Immunosorbent Assay (ELISA) as stated in the manufacturer’s protocol. The tissue homogenates were fixed in RIPA buffer with a protease inhibitor cocktail (Merck) and incubated at -20°C. The level of XCL1 was measured in the tissue homogenates using the Mouse XCL1/Lymphotactin ELISA Kit (Sandwich ELISA, LS-F53223; LifeSpan Biosciences) with the following detection ranges: 6.25–400 pg/ml. The manufacturer provided the positive controls for each assay.

#### 2.5.4 Immunofluorescence analysis by confocal microscopy

Seven days after CCI, the mice were sacrificed, and their spinal cords were removed and postfixed in 4% paraformaldehyde (PFA) overnight at 4°C. After dehydration, the tissues were paraffin embedded and sectioned (7 μM) on a microtome (Leica, RM45). Adjacent coronal sections from corresponding regions of the lumbar (L4 to L6) spinal cords of naive and CCI mice were incubated overnight at 4°C with the following primary antibodies: rabbit anti-XCL1 (1:50, Novus Biologicals), rabbit anti-ITGA9 (1:50, Abcam), rabbit anti-XCR1 (1:50, Lifespan Biosciences), mouse anti-NeuN (1:250, Merck), rat anti-IBA1 (1:1000, Abcam), and chicken anti-GFAP (1:10000, Merck). Antigen-bound primary antibodies were visualized with appropriate Alexa Fluor 488/594–conjugated donkey secondary antibodies (1:100, Invitrogen). Hoechst 33342 (Invitrogen) was used to stain cell nuclei. Stained sections were examined and acquired under a high-class confocal microscope (Leica TCS SP8 WLL) equipped with HyD, PMT and TLD detectors. The ipsilateral part of the lumbar spinal cord was visualized on representative images.

#### 2.5.5 Statistical analysis

The behavioral studies (*in vivo*) are presented as the means ± SEMs. The biochemical studies (*ex vivo*) are presented as fold changes relative to the controls (naive) ± SEM. The RT−qPCR results are presented as the normalized averages derived from the threshold cycle. The results of i.t. administration of YA4/vMIP-II in CCI-induced neuropathy (mean ± SEM) were statistically evaluated using a t test with Welsh correction. The other results (mean ± SEM) were evaluated using one-way ANOVA (F value) followed by Bonferroni’s *post hoc* test for comparison of intergroup differences (p value). Additionally, the results were evaluated using two-way ANOVA (F value) to determine the time × drug interaction. All of the statistical analyses mentioned above were performed with GraphPad Prism ver. 8.1.1 (330) (GraphPad Software, Inc., San Diego, USA).

## 3 Results

### 3.1 Spatiotemporal changes in the mRNA and/or protein levels of XCL1, its receptors and pain-related behavior after chronic constriction injury of the sciatic nerve in mice

**Figure 1:**
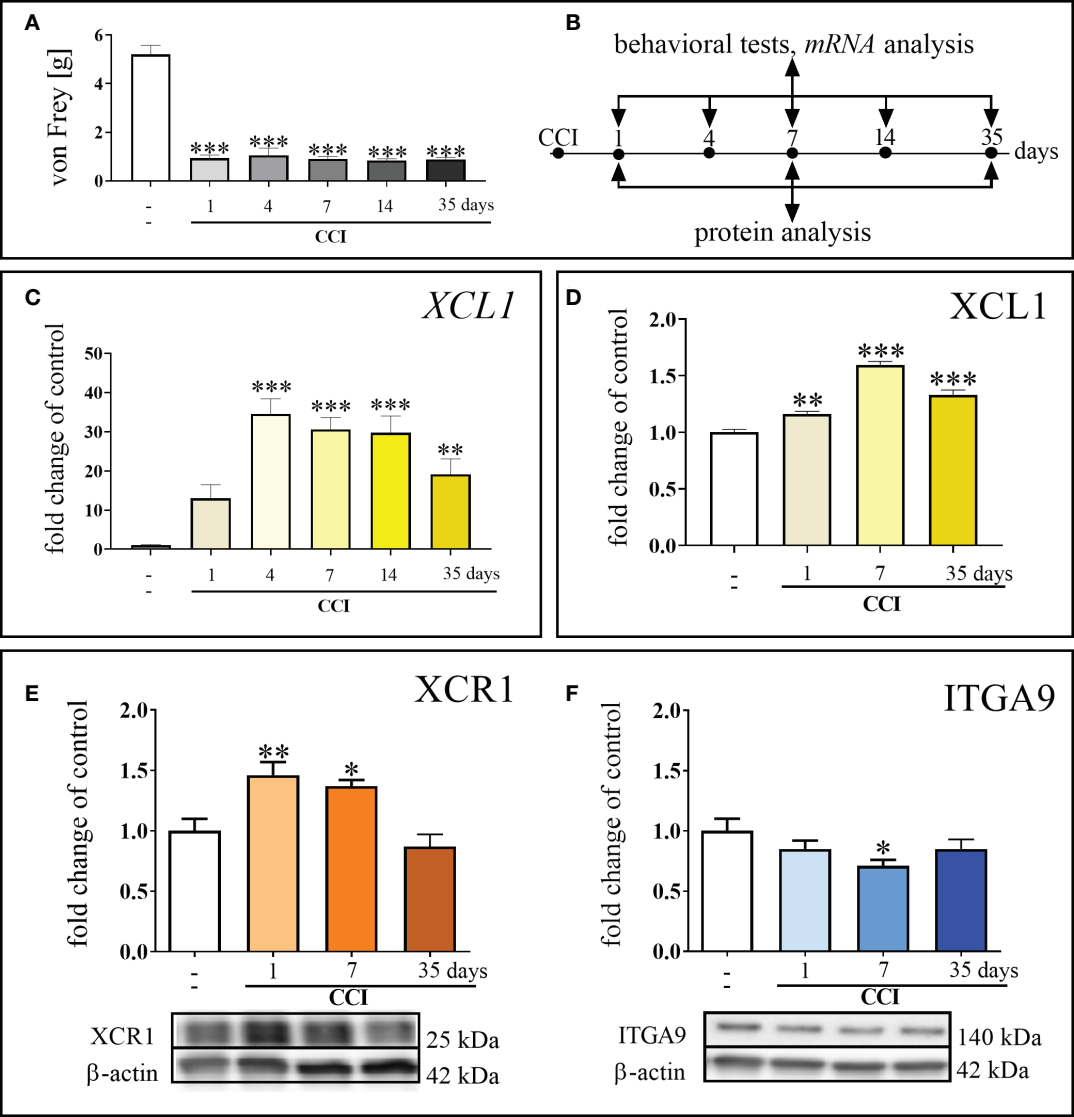
Development of mechanical hypersensitivity after chronic constriction injury (CCI) of the sciatic nerve in mice (1, 4, 7, 14, 35 days) **(A)**. Scheme of tissue collection at the indicated time points for behavioral tests and *mRNA/p*rotein analyses **(B)**. Time-dependent changes in the expression of XCL1 *mRNA* by RT–qPCR **(C)** and protein by ELISA **(D)**; XCR1 protein by Western blot **(E)** and ITGA9 protein by Western blot **(F)** in the spinal cord of naive and chronic constriction injury-exposed mice (1, 4, 7, 14 and/or 35 days). The data are presented as the mean fold changes relative to the control ± SEM (n =5–10). The results were evaluated using one–way ANOVA followed by Bonferroni’s *post hoc* test for comparisons of selected pairs. ^✽^p < 0.05; ^✽✽^p < 0.01; ^✽✽✽^p < 0.001 indicate significant differences between the naive vs. CCI-exposed groups at each of the investigated time points: 1, 4, 7, 14, 35 days. “-”– naive.

Chronic constriction injury led to the development of mechanical [F = 70.90; p < 0.0001] (**Figures 1A, B**) hypersensitivity. These pain-related changes were observed until the last time point tested, as shown using the von Frey test. The mRNA level of *XCL1* was significantly elevated 4 days after CCI [F = 9.492; p < 0.0001], and this elevated level was maintained until the 35th day after nerve injury [F = 9.492; p = 0.0033] (**Figure 1C**). In the protein study, the elevated level of XCL1 protein was maintained from 1 day after CCI [F = 70.26; p = 0.0011] up to day 35 [F = 70.26; p < 0.0001] (**Figure 1D**). The protein level of XCR1 increased significantly 1 day after surgery [F = 9.88; p = 0.0031] and remained elevated until day 7 in the spinal cord [F = 9.88; p = 0.0102] (**Figure 1E**). The protein level of ITGA9 was significantly reduced compared to that in naive animals on day 7 after damage to the sciatic nerve [F = 2.37; p = 0.0153] (**Figure 1F**).

### 3.2 Effects of a single intrathecal XCL1 nAb administration on pain-related behavior measured 7 days after chronic constriction injury of the sciatic nerve in mice

**Figure 2:**
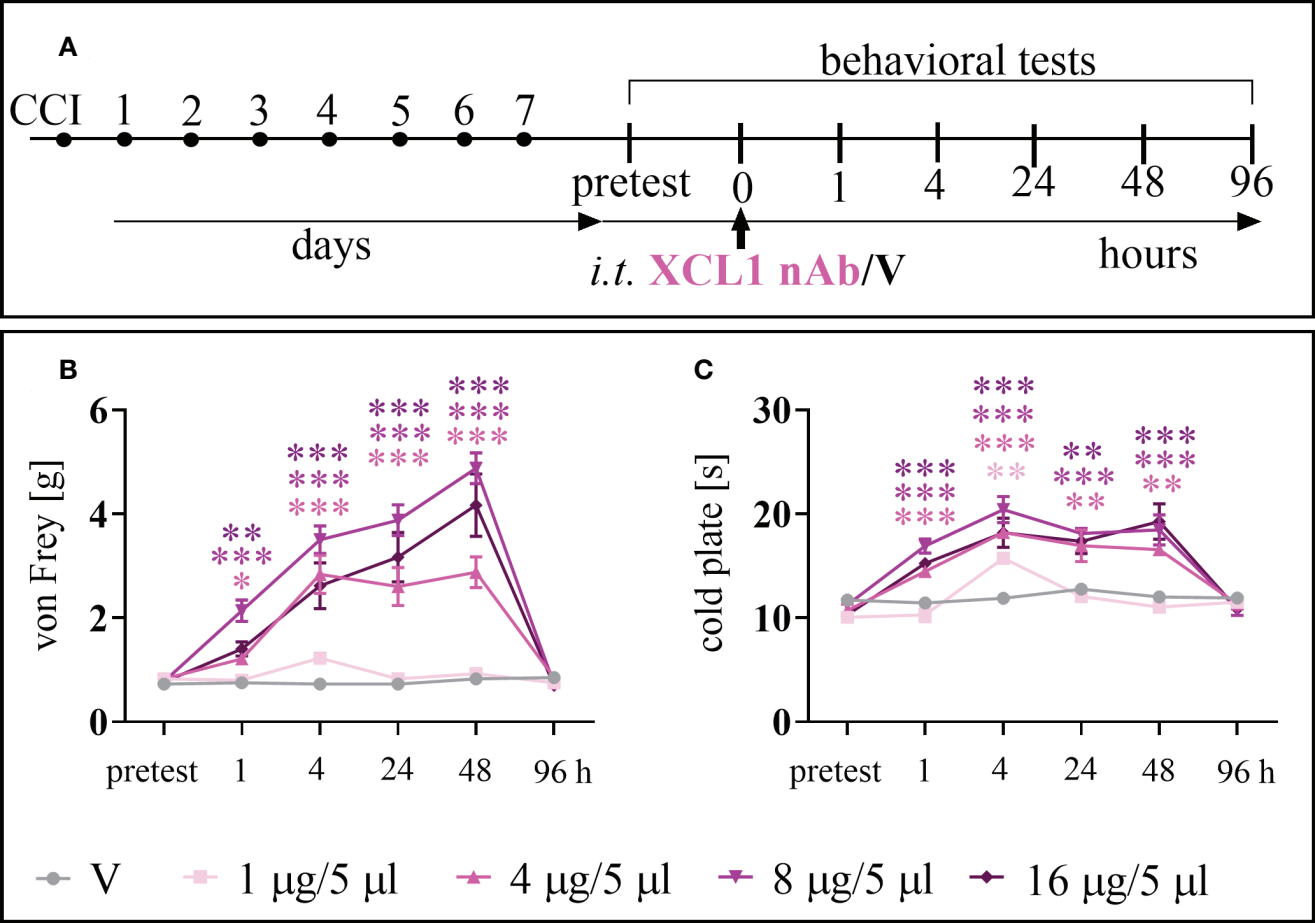
The effects of chemokine-C-motif ligand 1 (XCL1) neutralizing antibody (nAb) administered according to scheme **(A)**, at a dose of 1, 4, 8, or 16 μg/5 μl, on mechanical **(B)** and thermal **(C)** hypersensitivity 7 days after chronic constriction injury (CCI) of the sciatic nerve in mice. The data are presented as the mean ± SEM (n =6–8). The results were evaluated using one–way ANOVA followed by Bonferroni’s *post hoc* test for comparisons of selected pairs. ^✽^p < 0.05; ^✽✽^p < 0.01; ^✽✽✽^p < 0.001 indicate significant differences between the V– vs. nAb–treated groups at each of the investigated time points: 1, 4, 24, 48 and 96 h. “V”– vehicle (PBS).

The analgesic effect of XCL1 nAb administration (**Figure 2A**) in CCI-exposed mice was observed for doses of 1, 4, 8, and 16 μg/5 μl in the von Frey (**Figure 2B**) and/or cold plate (**Figure 2C**) tests. The dose of 1 μg/5 μl was effective 4 hours after administration [cold plate: F = 15.04; p = 0.0025]. The doses of 4, 8, 16 μg/5 μl showed their analgesic properties 1 hour after administration (4 μg/5 μl [von Frey: F = 20.93; p = 0.0117; cold plate: F = 26.51; p = 0.0003], 8 μg/5 μl [von Frey: F = 20.93; p < 0.0001; cold plate: F = 26.51 p < 0.0001], 16 μg/5 μl [von Frey: F = 20.93; p = 0.0015; cold plate: F = 26.51 p < 0.0001]), and their effects were elevated until 48 hours (4 μg/5 μl [von Frey: F = 40.02; p < 0.0001; cold plate: F = 14.47; p = 0.0019], 8 μg/5 μl [von Frey: F = 40.02 p < 0.0001; cold plate: F =14.47; p < 0.0001], 16 μg/5 μl[von Frey: F = 40.02 p < 0.0001; cold plate: F = 14.47; p < 0.0001]). Two-way ANOVA confirmed a significant interaction between the treatment and the analyzed time points [von Frey: F =14.39; cold plate: F = 6.56].

### 3.3 Effects of a single intrathecal XCL1 nAb administration on morphine and buprenorphine analgesia 7 days after chronic constriction injury of the sciatic nerve in mice

**Figure 3:**
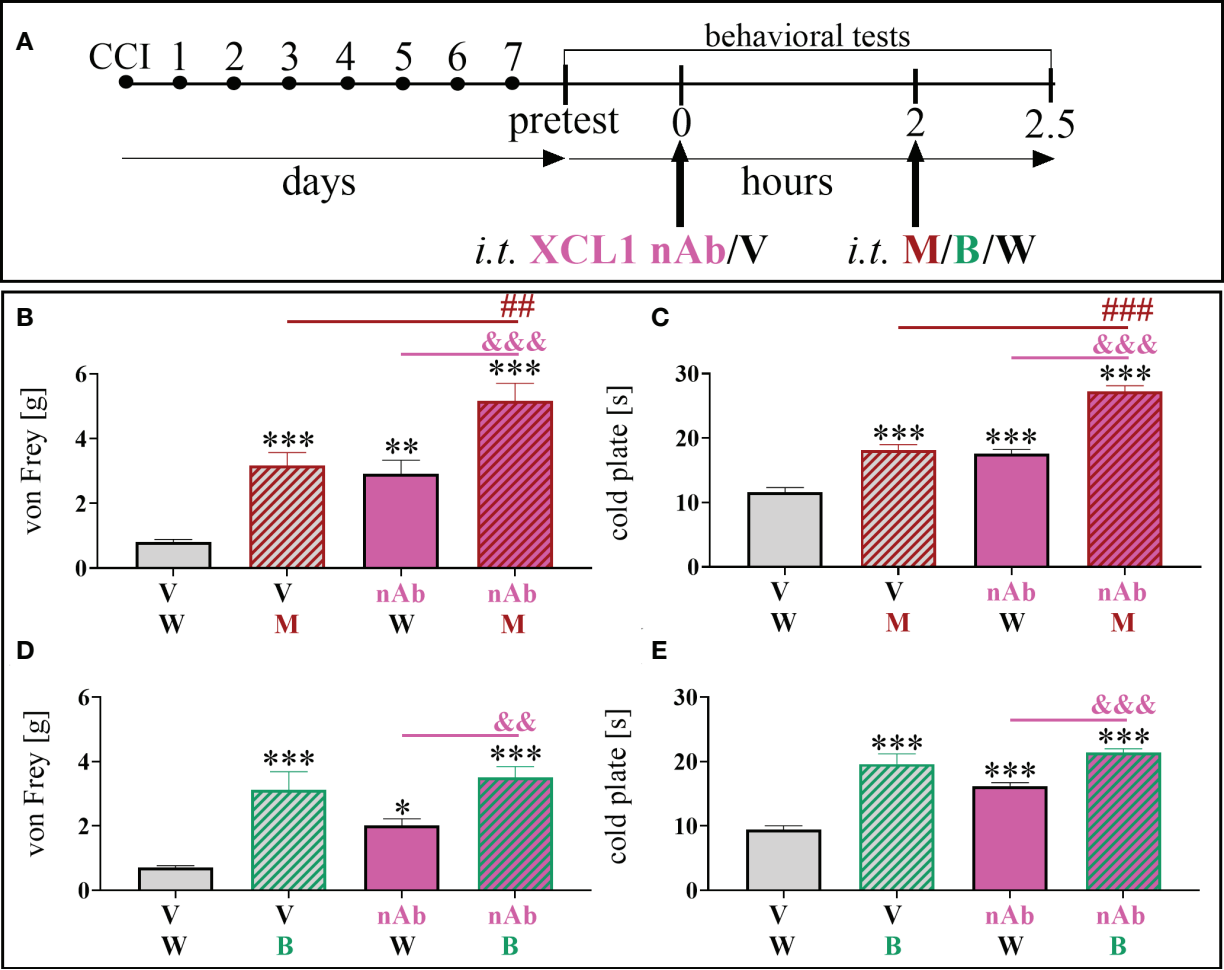
The influence of a chemokine-C-motif ligand 1 (XCL1) neutralizing antibody (nAb) **(B–E)** at a dose of 8 μg/5 μl on morphine (M) 2.5 μg/5 μl **(B, C)** and buprenorphine **(B)** 2.5 μg/5 μl **(D, E)** effectiveness, administered according to scheme **(A)**, 7 days after chronic constriction injury (CCI) of the sciatic nerve in mice. The data are presented as the mean ± SEM (n =6). The results were evaluated using one–way ANOVA followed by Bonferroni’s *post hoc* test for comparisons of selected pairs. ^✽^p < 0.05; ^✽✽^p < 0.01; ^✽✽✽^p < 0.001 indicate significant differences between the V+W– and M–/B–/nAb–treated groups; ^##^p < 0.01; ^###^P < 0.001 indicate significant differences between the M– and M+nAb–treated groups; ^&&^p < 0.01; ^&&&^P < 0.001 indicate significant differences between the nAb– and M+nAb–/B+nAb–treated groups. “V”– vehicle (PBS); “W” – vehicle (water for injections).

For the co-administration with opioids (**Figure 3A**) we have chosen the dose of 8 μg/5 μl, basing on its the effectiveness as shown in time-/dose- dependency study (**Figure 2**). Selected dose of XCL1 nAb (8 μg/5 μl) and morphine (2.5 μg/5 μl) similarly significantly reduced mechanical [F = 20.02; p = 0.0004] (**Figure 3B**) and thermal [F = 66.85; p < 0.0001] hypersensitivity (**Figure 3C**). Buprenorphine at a dose of 2.5 μg/5 μl also diminished both mechanical [F = 13.07; p < 0.0001] (**Figure 3D**) and thermal [F = 31.43; p < 0.0001] (**Figure 3E**) hypersensitivity in CCI-exposed mice.

The influence of XCL1 nAb on morphine analgesia was significant and reduced both mechanical [F = 20.02; p = 0.0020] (**Figure 3B**) and thermal [F = 66.85; p < 0.0001] (**Figure 3C**) hypersensitivity compared to morphine administered alone. Otherwise, there was no observable impact of the XCL1 nAb on buprenorphine analgesia (**Figures 3D, E**).

### 3.4 Effects of chronic intraperitoneal minocycline administration on mechanical hypersensitivity and changes in the protein levels of XCL1-, XCR-1-, ITGA9-, IBA1-, and GFAP-positive cells 7 days after chronic constriction injury of the sciatic nerve in mice

**Figure 4:**
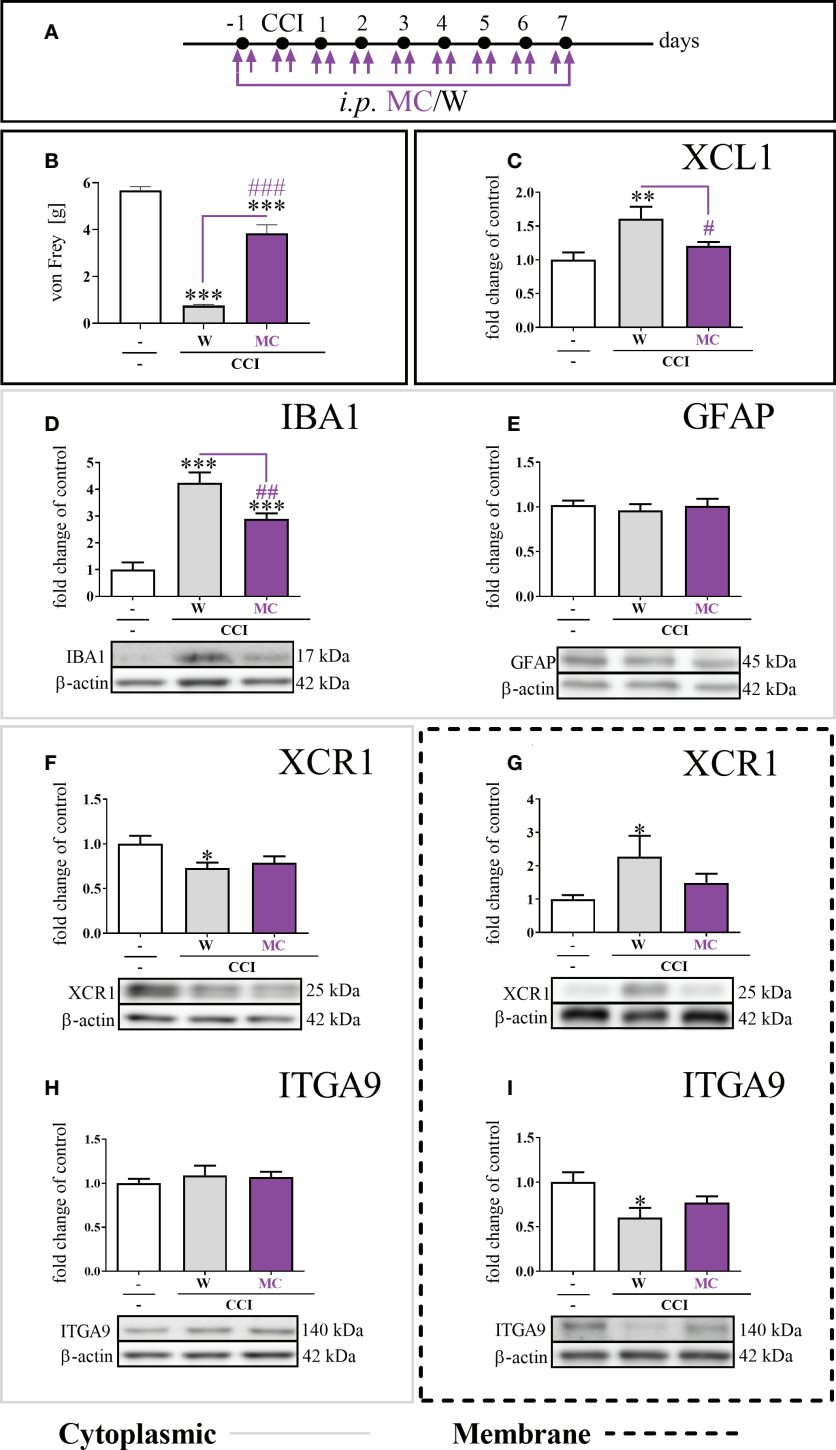
The influence of repeated (preemptive and then twice daily for 7 days) minocycline (MC) administration **(A)**, at a dose of 30 mg/kg, on mechanical hypersensitivity **(B)**; XCL1 protein level in the cytoplasmic fraction by ELISA **(C)**, IBA1 **(D)**, GFAP **(E)**, XCR1 **(F)**, and ITGA9 **(H)** protein levels in the cytoplasmic fraction by Western blot; XCR1 **(G)** and ITGA9 **(I)** protein levels in the membrane fraction by Western blot, seven days after chronic constriction injury (CCI) of the sciatic nerve in mice. The data are presented as the mean fold changes relative to the control ± SEM (n =5–14). The results were evaluated using one-way ANOVA followed by Bonferroni’s *post hoc* test for comparisons of selected pairs; ^✽^p < 0.05; ^✽✽^p < 0.01; ^✽✽✽^p < 0.001 indicate significant differences between the naive vs. W–/MC–treated groups; ^#^p < 0.05; ^##^p < 0.01; ^###^p < 0.001 indicate significant differences between the W– vs. MC–treated groups. “-”– naive; “W” – vehicle (water for injections).

Chronic minocycline administration (**Figure 4A**) significantly reduced mechanical hypersensitivity, which had been fully developed in W–treated animals 7 days after CCI [F = 87.26; p < 0.0001] (**Figure 4B**). The study revealed that after chronic MC treatment, there was a significant reduction in the protein levels of XCL1 [F = 6.43; p = 0.0326] (**Figure 4C**) and IBA1 [F = 31.09; p = 0.0082] (**Figure 4D**) compared to CCI-exposed mice. There were no changes in the level of GFAP (**Figure 4E**).

The level of the XCR1 protein in the cytoplasmic fraction was diminished [F = 3.37; p = 0.0252] in the W–treated group, and MC did not influence this effect (**Figure 4F**). The protein level of XCR1 in the membrane fraction was significantly elevated (**Figure 4G**) in the W–treated group [F = 3.63; p = 0.0159]. It was different in the group receiving MC, where the level of the XCR1 protein in the membrane fraction was not changed compared to naive animals (**Figure 4G**). Regarding the ITGA9 protein, the expression levels were not changed between the W–and MC–treated groups in the cytoplasmic fraction (**Figure 4H**). The protein level of ITGA9 in the membrane fraction in the CCI-exposed group was significantly diminished [F = 3.96; p = 0.0115] (**Figure 4I**). This was not the case in the group receiving MC, in which the level of the ITGA9 protein in the membrane fraction was not changed compared to that in naive animals (**Figure 4I**). Additionally, the minocycline treatment diminished the levels of p38, ERK, JNK and AKT (**Supplementary File – Figure 1**).

### 3.5 Effects of a single intrathecal ITGA9 nAb (YA4) and XCR1 antagonist (vMIP-II) administration preceded by XCL1 injection in naive mice on mechanical and thermal hypersensitivity

**Figure 5:**
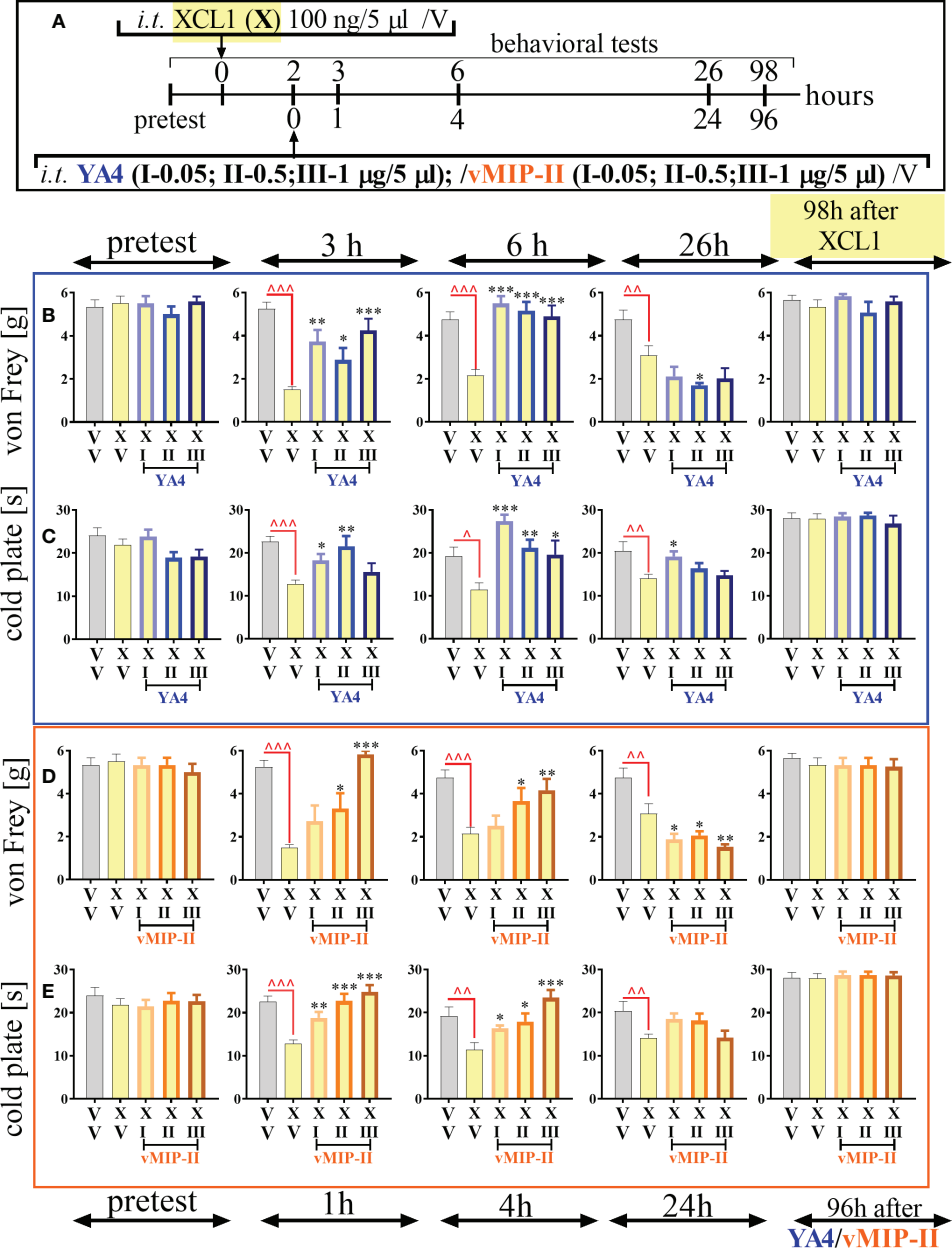
The influence of ITGA9 neutralization by YA4 **(B, C)** or XCR1 blockade by vMIP-II **(D, E)**, according to scheme **(A)**, at a dose of 0.05 (I), 0.5 (II), 1 (III) μg/5 μl on mechanical **(B, D)** and thermal **(C, E)** hypersensitivity in naive mice after pronociceptive XCL1 100 ng/5 μl (X) administration. Data are presented as the mean ± SEM (n =4–6). The results were evaluated using one-way ANOVA followed by Bonferroni’s *post hoc* test for comparisons of selected pairs; ^p < 0.05; ^^p < 0.01; ^^^p < 0.001 indicate significant differences between the veh-(V+V) vs. XCL1(X+V)–treated group; ^✽^p < 0.05; ^✽✽^p < 0.01; ^✽✽✽^p < 0.001 indicate significant differences between the XCL1(X+V)– vs. XCL1+YA4–/XCL1+vMIP-II–treated groups. Abbreviations: “V”– vehicle (PBS).

ITGA9 neutralization by YA4 diminished mechanical (**Figure 5B**) and thermal (**Figure 5C**) hypersensitivity developed after pronociceptive XCL1 administration (XCL1 was injected 2 hours before YA4) in naive animals (**Figure 5A**). The effect was observed for the XCL1+YA4-treated groups compared to the XCL1+V-treated group in both behavioral tests since 1 hour (3 hours after XCL1 administration) for doses of 0.05 μg/5 μl [von Frey: F = 9.94; p = 0.0018; cold plate: F = 5.90; p = 0.0298], 0.5 μg/5 μl [von Frey: F = 9.94; p = 0.0402; cold plate: F = 5.90; p = 0.0011], and 1 μg/5 μl [von Frey F = 9.94; p = 0.0002]. It was still effective until the 4th hour (6th hour after XCL1 administration) for doses of 0.05 μg/5 μl [von Frey: F = 11.95; p < 0.0001; cold plate: F = 6.85; p < 0.0001], 0.5 μg/5 μl [von Frey: F = 11.95 p < 0.0001; cold plate: F = 6.85; p = 0.0041], and 1 μg/5 μl [von Frey: F = 11.95 p < 0.0001; cold plate: F = 6.85; p = 0.0135]. Twenty-four hours after administration of YA4 (26 hours after XCL1 administration), there was still an observable antinociceptive effect in the cold plate test [dose of 0.05 μg/5 μl: F = 3.82; p = 0.0164] (**Figures 5B, C**).

The analgesic effect of XCR1 blockade by vMIP-II injection was more dose-dependent and diminished mechanical (**Figure 5D**) and thermal (**Figure 5E**) hypersensitivity - the pronociceptive effect of XCL1 administration (XCL1 was administered 2 hours before vMIP-II) in naive animals (**Figure 5A**). The effect was observed for the XCL1+ vMIP-II-treated groups compared to the XCL1+V-treated group in both behavioral tests after 1 hour (3 hours after XCL1 administration) for doses of 0.05 μg/5 μl [cold plate: F = 12.10; p = 0.0046], 0.5 μg/5 μl [von Frey: F = 13.72; p = 0.0139; cold plate: F = 12.10; p < 0.0001], and 1 μg/5 μl [von Frey: F = 13.72; p < 0.0001; cold plate: F = 12.10; p < 0.0001]. It was still effective until the 4th hour (6th hour after XCL1 administration) for doses of 0.05 μg/5 μl [cold plate: F = 6.72; p = 0.0494], 0.5 μg/5 μl [von Frey: F = 5.59; p = 0.0293; cold plate: F = 6.72; p = 0.0119], and 1 μg/5 μl [von Frey: F = 5.59; p = 0.0051; cold plate: F = 6.72; p < 0.0001] (**Figures 5D, E**).

### 3.6 Effects of a single intrathecal ITGA9 nAb (YA4) and XCR1 antagonist (vMIP-II) administration on mechanical and thermal hypersensitivity 7 days after chronic constriction injury of the sciatic nerve in mice

**Figure 6:**
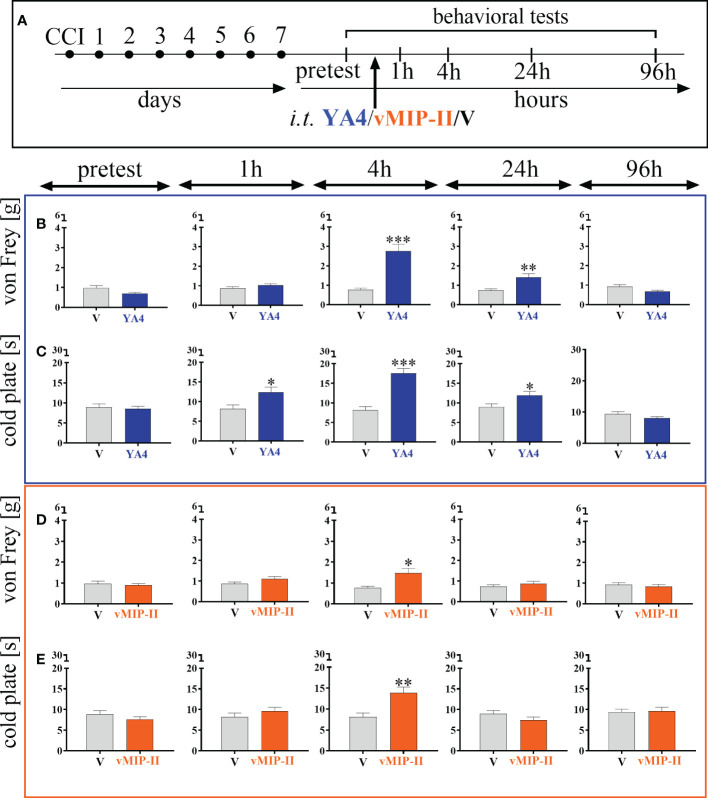
The influence of ITGA9 neutralization by YA4 **(B, C)** or XCR1 blockade by vMIP-II **(D, E)**, administered according to scheme **(A)**, at a dose of 1 μg/5 μl on mechanical **(B, D)** and thermal **(C, E)** hypersensitivity 7 days after chronic constriction injury (CCI) of the sciatic nerve in mice. Data are presented as the mean ± SEM (n =5–8). The results were evaluated using t tests for comparisons of selected pairs; ^✽^p < 0.05; ^✽✽^p < 0.01; ^✽✽✽^p < 0.001 indicate significant differences between the V– vs. YA4–/vMIP-II–treated groups. “V”– vehicle (PBS).

The neutralization of ITGA9 by YA4 (**Figure 6A**) at a dose of 1 μg/5 μl started to influence mechanical hypersensitivity and simultaneously was the most effective 4 hours after administration [t = 5.58; p  = 0.0006] and lasted until 24 hours [t = 3.22; p  = 0.0097] (**Figure 6B**). Similarly, in the case of thermal hypersensitivity, neutralization started to be effective 1 hour after administration [t = 2.72; p  = 0.0190], was the most powerful 4 hours after administration [t = 6.17; p < 0.0001], and similarly ceased but was still effective at 24 hours [t = 2.35; p  = 0.0366] (**Figure 6C**).

The blockade of XCR1 by vMIP-II injection (**Figure 6A**) at a dose of 1 μg/5 μl also effectively diminished mechanical and thermal hypersensitivity in the CCI model, but only 4 hours after administration, as revealed both by the von Frey test [t = 3.06; p  = 0.0207] (**Figure 6D**) and the cold plate test [t = 3.56; p  = 0.0042] (**Figure 6E**).

### 3.7 Effects of a single intrathecal ITGA9 nAB (YA4) and XCR1 antagonist (vMIP-II) administration on morphine and buprenorphine analgesia 7 days after chronic constriction injury of the sciatic nerve in mice

**Figure 7:**
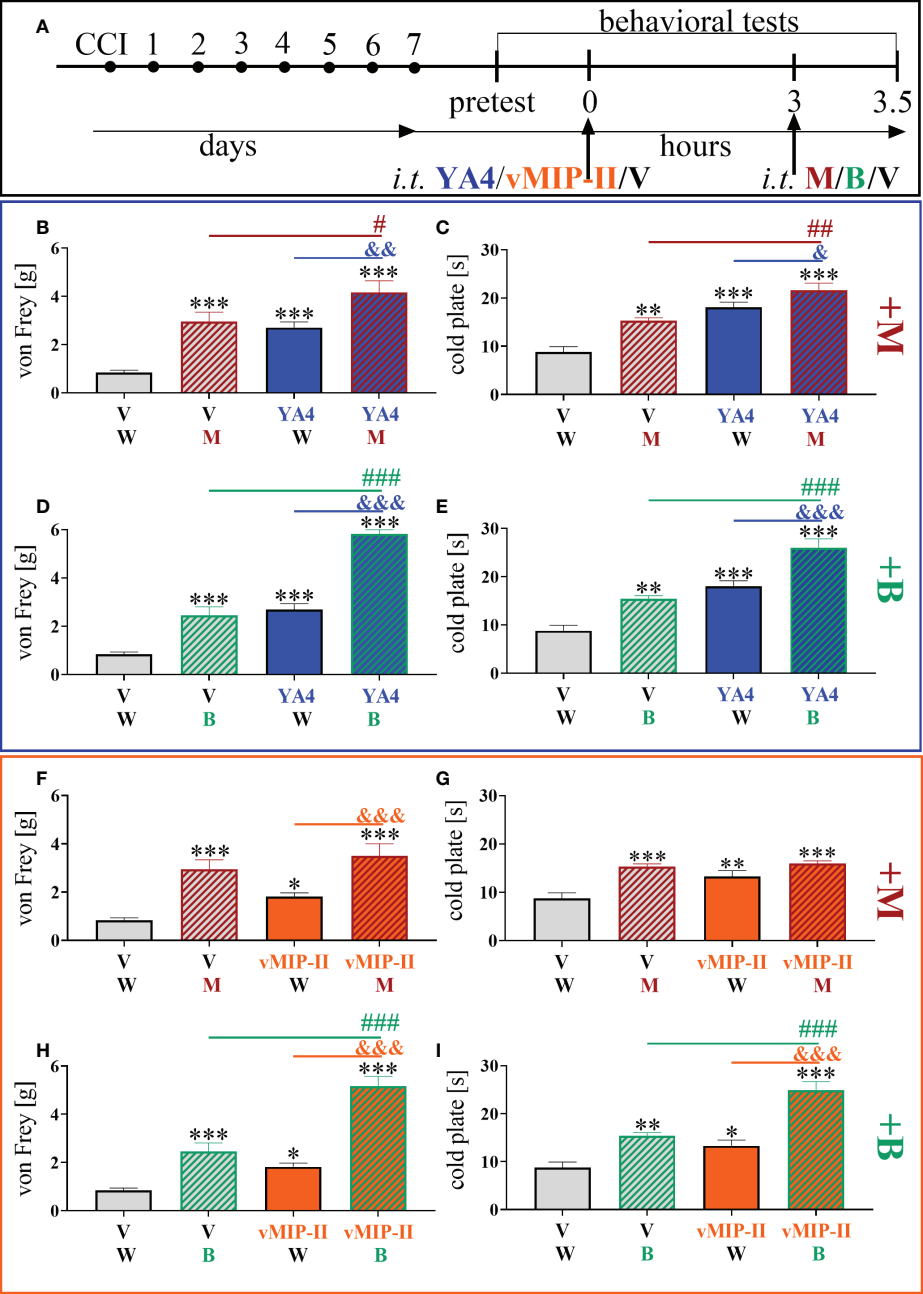
The influence of ITGA9 neutralization by YA4 **(B–E)** or XCR1 blockade by vMIP-II **(F–I)** at a dose of 1 μg/5 μl on morphine (M) 2.5 μg/5 μl **(B, C, F, G)** and buprenorphine **(B)** 2.5 μg/5 μl **(D, E, H, I)** effectiveness, administered according to scheme **(A)**, 7 days after chronic constriction injury (CCI) of the sciatic nerve in mice. Data are presented as the mean ± SEM (n =5–12). The results were evaluated using one-way ANOVA followed by Bonferroni’s *post hoc* test for comparisons of selected pairs; ^✽^p < 0.05; ^✽✽^p < 0.01; ^✽✽✽^p < 0.001 indicate significant differences between the V– vs. YA4–/vMIP-II–/M–/B–treated groups; ^#^p < 0.05; ^##^p < 0.01; ^###^P < 0.001 indicate significant differences between the M–/B–treated vs. M+YA4–/B+YA4–/B+vMIP-II–treated groups; ^&^p < 0.05; ^&&^p < 0.01; ^&&&^p < 0.001 indicate significant differences between the YA4– vs. M+YA4–/B+YA4– or vMIP-II– vs. M+vMIP-II–/B+vMIP-II–treated groups. “V”– vehicle (PBS); “W” – vehicle (water for injections).

The influence of YA4 on morphine analgesia (**Figure 7A**) was significant and more effectively reduced both mechanical [F = 13.27; p = 0.0239] (**Figure 7B**) and thermal [F = 15.54; p = 0.0025] (**Figure 7C**) hypersensitivity compared to the administration of substances alone. The influence of YA4 on buprenorphine effectiveness (**Figure 7A**) was also observable. Strong reduction of both mechanical [F = 50.06; p < 0.0001] (**Figure 7D**) and thermal [F = 23.16; p < 0.0001] (**Figure 7E**) hypersensitivity was demonstrated compared to separately injected compounds.

After administration of vMIP-II with buprenorphine (**Figure 7A**), attenuation of thermal [F = 24.00; p < 0.0001] (**Figure 7I**) and mechanical [F = 42.60; p < 0.0001] (**Figure 7H**) hypersensitivity was observed and was the strongest compared to single substance action. Otherwise, there was no observable impact of vMIP-II on morphine analgesia in either behavioral test (**Figures 7F, G**).

### 3.8 The cellular localization of XCL1, XCR1 and ITGA9 in the spinal cord 7 days after chronic constriction injury of the sciatic nerve in mice revealed by immunofluorescence staining

**Figure 8:**
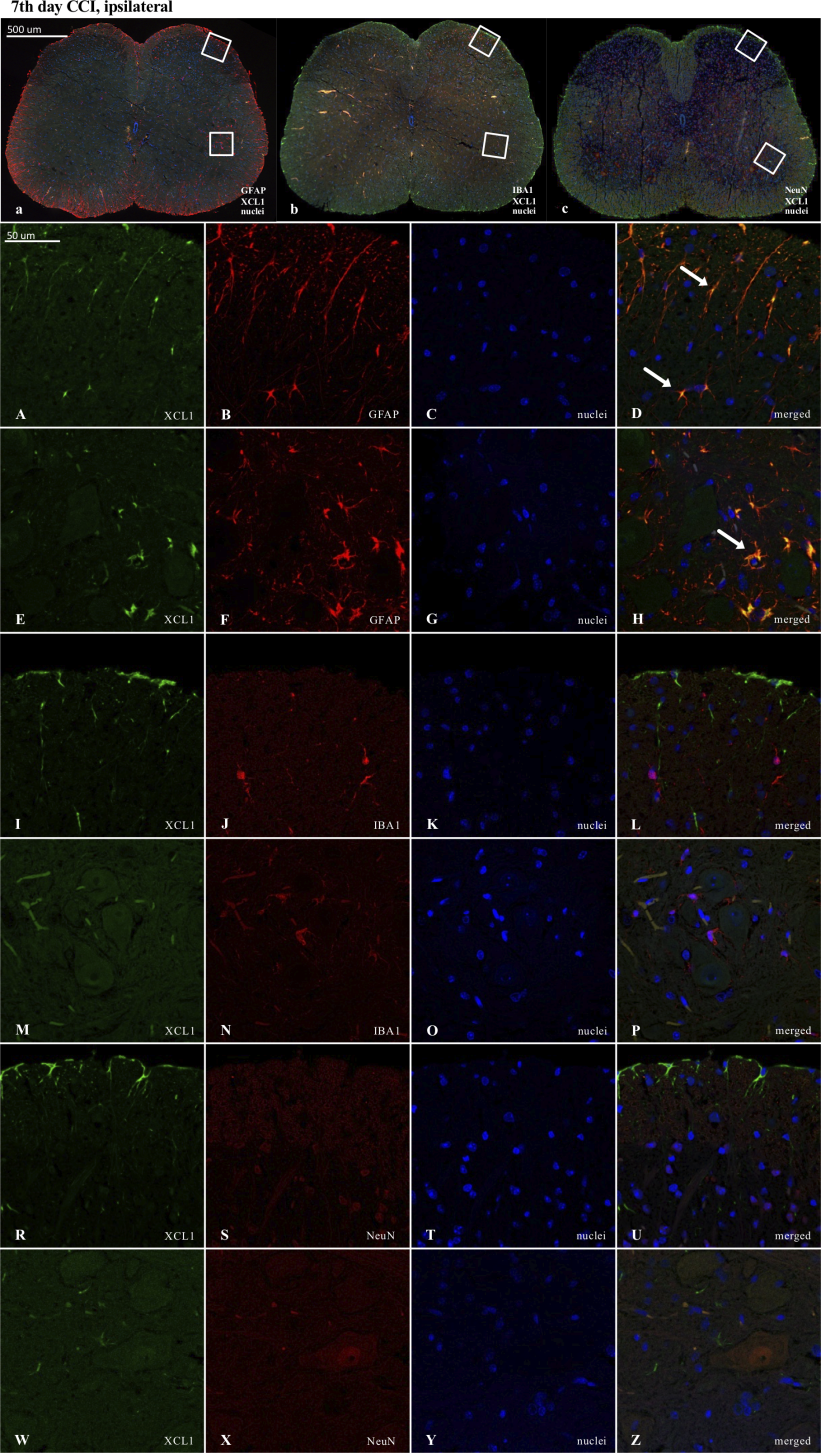
Immunofluorescence analysis of chemokine-C-motif ligand 1 (XCL1) localization in the lumbar (L4 to L6) spinal cord 7 days after chronic constriction injury (CCI) of the sciatic nerve in mice. Dorsal **(A–D, I–L, R–U)** and ventral **(E–H, M–P, W–Z)** parts of lumbar spinal cord (**a,b,c**) were shown as an approximate fragments of selected images. Representative immunofluorescence images from colocalization analysis performed on spinal cord, paraffin-embedded 7 μM microtome slices: XCL1 (green: **A, E, I, M, R, W**) with astroglia marker glial fibrillary acidic protein; (GFAP, red: **B, F**); microglia marker ionized calcium-binding adaptor molecule 1; (IBA1, red: **J, N**); and with neuronal marker neuronal nucleus; (NeuN, red: **S, X**), Nuclei are in blue (**C, G, K, O, T, Y**). High magnification, three-dimensional image rendering shows XCL1 localization inside GFAP-positive cells (yellow: **D, H**). Scale bars: 50 μm (**a, b, c**), 500 μm **(A–Z)**.

**Figure 9:**
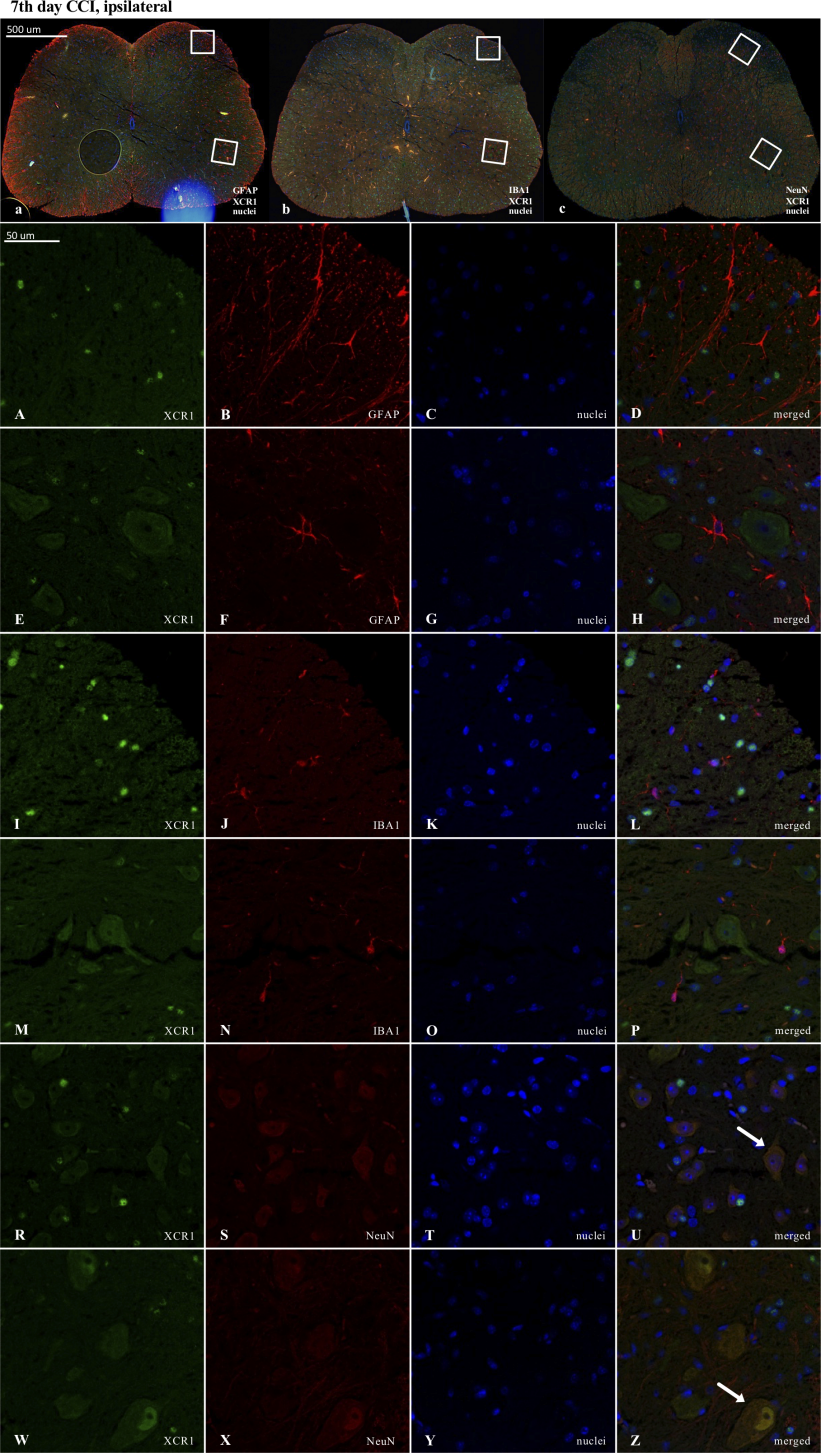
Immunofluorescence analysis of chemokine-C-motif receptor 1 (XCR1) localization in the lumbar (L4 to L6) spinal cord 7 days after chronic constriction injury (CCI) of the sciatic nerve in mice. Dorsal **(A–D, I–L, R–U)** and ventral **(E–H, M–P, W–Z**) parts of lumbar spinal cord (**a,b,c**) were shown as an approximate fragments of selected images. Representative immunofluorescence images from colocalization analysis performed on spinal cord, paraffin-embedded 7 μM microtome slices: XCR1 (green: **A, E, I, M, R, W**) with astroglia marker glial fibrillary acidic protein; (GFAP, red: **B,F**); microglia marker ionized calcium-binding adaptor molecule 1; (IBA1, red: **J, N**); and with neuronal marker neuronal nucleus; (NeuN, red: **S, X**), Nuclei are in blue (**C, G, K, O, T, Y**). High magnification, three-dimensional image rendering shows XCR1 localization inside NeuN-positive cells (yellow: **U, Z**). Scale bars: 50 μm (**a, b, c**), 500 μm **(A–Z)**.

**Figure 10:**
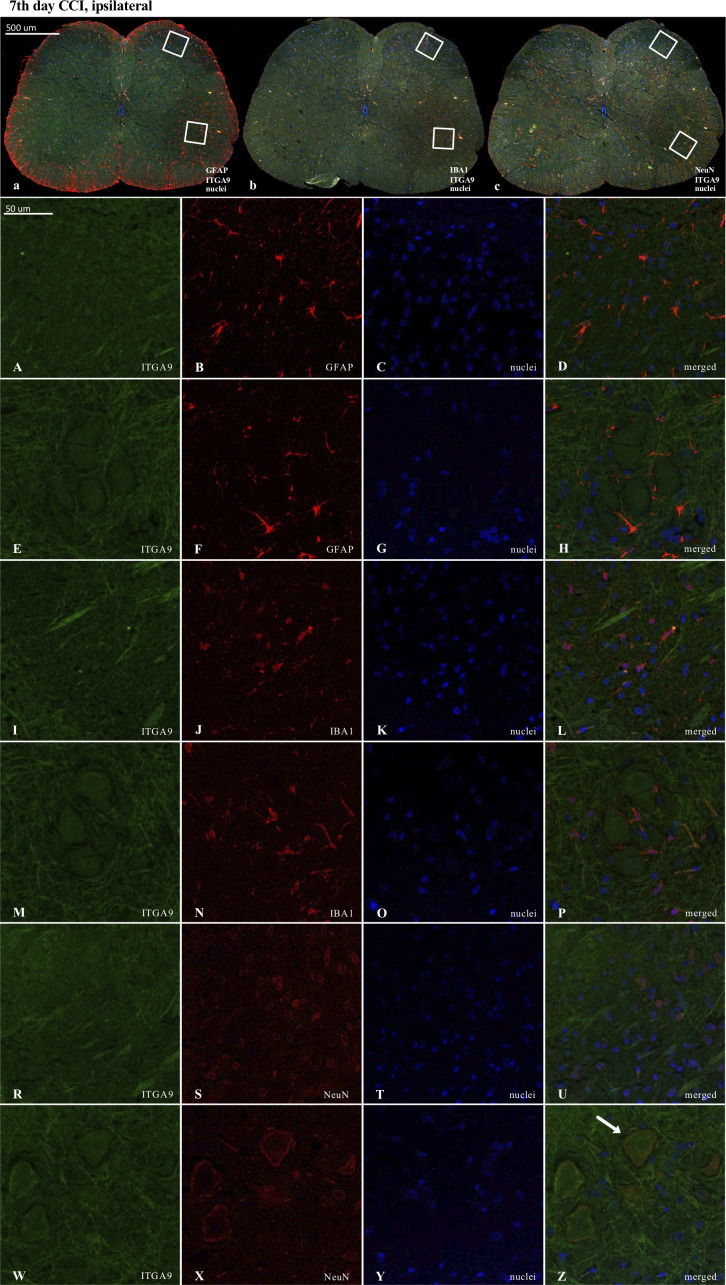
Immunofluorescence analysis of integrin alpha-9 (ITGA9) localization in the lumbar (L4 to L6) spinal cord 7 days after chronic constriction injury (CCI) of the sciatic nerve in mice. Dorsal **(A–D, I–L, R–U)** and ventral **(E–H, M–P, W–Z**) parts of lumbar spinal cord (**a,b,c**) were shown as an approximate fragments of selected images. Representative immunofluorescence images from colocalization analysis performed on spinal cord, paraffin-embedded 7 μM microtome slices: ITGA9 (green: **A, E, I, M, R, W**) with astroglia marker glial fibrillary acidic protein; (GFAP, red: **B, F**); microglia marker ionized calcium-binding adaptor molecule 1; (IBA1, red: **J, N**); and with neuronal marker neuronal nucleus; (NeuN, red: **S, X**), Nuclei are in blue (**C, G, K, O, T, Y**). High magnification, three-dimensional image rendering shows ITGA9 localization inside NeuN-positive cells (yellow: **U, Z**). Scale bars: 50 μm (**a, b, c**), 500 μm **(A–Z)**.

Results of the immunofluorescence analysis from CCI-exposed mice are shown in Figures: 8A-Z, 9A-Z, 10A-Z and from naive animals in the **Supplementary File: Figures 2A-Z, 3A-Z, 4A-Z**. For robustness of the visualization, two independent corresponding regions of the spinal cord of each section were compared, as shown in **Figures 8a–c**, **Figures 9a–c** and **Figures 10a–c**.

Fluorescence immunohistochemical staining revealed clear colocalization of XCL1 with GFAP-positive cells (**Figures 8D, H**). No colocalization was observed between XCL1 and IBA1 (microglia marker) or NeuN (neuronal marker) (**Figures 8L, P, U, Z**; respectively).

In contrast, in the case of XCR1, there was clear colocalization with NeuN-positive cells (**Figures 9U, Z**). However, it is worth emphasizing that a strong XCR1-positive signal was observed in other cells but not in cells expressing GFAP (astroglia), IBA1 (microglia, macrophages), or NeuN (neurons) (**Figure 9**).

ITGA9 was also shown to colocalize with a neuronal marker (NeuN) (**Figures 10U, Z**) but similarly to XCR1, not with astroglia (**Figures 10D, H**) and IBA1-positive cells in the spinal cord (**Figures 10L, P**).

Because the activation of IBA1 and GFAP was expected to be visible in CCI-treated animals, the experiments designed to show colocalization were focused on these groups. Nevertheless, to maintain good laboratory practice, we also collected the stainings performed on naive animals (**Supplementary File – Figures 2–4**). As shown, after nerve injury, there was much stronger activation of IBA1-positive (microglia/macrophages) and GFAP-positive cells in the ipsilateral dorsal and ventral parts of the spinal cord (**Figures 8a, b**, **9a, b**, **10a, b**) than in the respective supplementary data (**Supplementary File – Figures 2a, b, 3a, b, 4a, b**).

## 4 Discussion

Our results demonstrated for the first time the upregulation in the level of XCL1 mRNA/protein during neuropathy development (already one day after CCI), which was maintained for up to 35 days, and as revealed by confocal analysis of immunofluorescent staining, these chemokines are produced mainly by spinal astroglial cells. Importantly, our study also unprecedentedly showed that blockade/neutralization of both XCL1 receptors, XCR1 by vMIP-II and ITGA9 by YA4, reverse XCL1 nociceptive properties. In addition, we proved that the XCL1 neutralizing antibody reduces mechanical and thermal hypersensitivity and improves morphine analgesia in CCI-exposed mice. Additionally, our results indicated that the mechanisms of minocycline analgesic action in neuropathy may also involve the decrease in the pronociceptive XCL1. Moreover, behavioral studies provided the first evidence that vMIP-II and YA4 diminish thermal and mechanical hypersensitivity in CCI-exposed mice and enhance buprenorphine and, in the case of YA4, also morphine analgesia. Importantly, immunofluorescence staining indicated that XCR1 and ITGA9 are expressed on neurons. Remarkably, our results clearly showed that blocking the XCL1-ITGA9 interaction appears to be more potent in relieving neuropathic pain.

XCL1 is known to play an essential role in the classical immune response (28). It is produced by subsets of T and NK cells during the course of inflammation, leading to chemotaxis of these cells by binding to XCR1 (28). However, in the course of neuropathy, the CD^4+^ and CD^8+^ T helper cells are unchanged in the spinal cord, in contrast to strongly activated glial cells (21). Recently, it was shown that the mRNA and protein level of XCL1 is increased in primary astroglia but not microglia in LPS-stimulated mouse cell cultures (27). Moreover, importantly, XCL1 stimulation of primary microglial and astroglial cells does not directly induce the production of pronociceptive interleukins (IL-1β, IL-18, IL-6) and chemokines (CCL3, CCL4, CCL9) (27). Therefore, we hypothesized that in neuropathy, XCL1 acts through neuronally localized receptors. Our immunofluorescence analysis proved that XCR1 and ITGA9 are located on spinal neurons, not micro- and astroglial cells. In the present study, we demonstrated for the first time the quick and strong upregulation of spinal XCL1, which lasts up to 5 weeks. Importantly, intrathecal injection of XCL1 in naive mice evoked thermal and mechanical hypersensitivity after 1 h, which lasted up to 24 h (7). Moreover, our studies provided the first evidence that neutralization of XCL1 results in reductions in thermal and mechanical hypersensitivity in CCI-exposed mice. In turn, XCL1-evoked hypersensitivity in naive mice is abolished by pretreatment with vMIP-II and YA4, suggesting that both receptors are responsible for its pronociceptive properties. Many studies have demonstrated that glial cells activated under neuropathic pain conditions produce many pronociceptive cytokines, including interleukins (23, 38, 39) and chemokines (21–24, 40–44). We have followed that concept, and based on our results, we propose that XCL1 is produced by astroglia and activates neuronal XCR1 and ITGA9, which are both strongly engaged in neuropathic pain development. It was already shown by an *in vitro* study that minocycline, a glial inhibitor, treatment before LPS stimulation prevented *XCL1* mRNA upregulation in primary astroglial cells (7). Our present results confirmed that consecutive minocycline treatment (twice daily, 7 days) attenuates CCI-evoked neuropathic pain by inhibiting microglia/macrophages and diminishing the levels of p38, ERK, JNK and AKT, which is congruent with literature data (13, 45–48). It was also demonstrated that minocycline diminished hypersensitivity of spinal neurons after traumatic spinal cord injury (49). Importantly, we observed for the first time that minocycline treatment also lowered the protein level of XCL1 in the CCI-induced neuropathy model, which might be considered behind the additional mechanism of its beneficial effects in neuropathy.

XCR1 is the well-known receptor for XCL1. It is expressed in T cells, B cells and neutrophils (50), Schwann cells, oligodendrocytes (51) and neurons (7, 51). Previous immunofluorescence studies have shown that in the nervous system, XCR1 is expressed in nonpeptidergic and non-IB4 binding terminals of A-delta and C-fiber afferents and/or within excitatory interneurons (51). Our immunofluorescence analysis of lumbar spinal cord sections showed that XCR1 is expressed by neurons and other cells but not microglia and astroglia. Importantly, the membrane, but not cytoplasmic, fraction of spinal protein level XCR1 increased after CCI but not in the group of animals receiving minocycline, which is probably related to its ability to prevent glial activation (52). Since minocycline causes a decrease in the level of XCL1, that is produced by activated astroglial cells, regulation of the membrane level of XCR1 may results from the ligand-receptor interactions favorably altered by it. The pharmacological blockade of XCR1 by vMIP-II was previously shown to inhibit the expression of pERK and p38MAPK in tissue exposed to XCL1 (51), which is a similar effect to that caused by minocycline treatment, as shown by our study and others (13, 14). Moreover, it lowers spontaneously hyperactive neuronal discharges, which are also characteristic of central sensitization (51). The behavioral analysis performed in our studies provided the first evidence that pharmacological blockade of XCR1 by vMIP-II may evoke neuropathic pain relief. These findings indicate that the XCL1/XCR1 axis can participate in many aspects of neuro-glial interactions and play a significant role in nociceptive transmission.

Importantly, XCL1 can also act through ITGA9 (30), an extracellular matrix component, which was shown to participate in the pathophysiology of some intractable disorders by the regulation of the cell physiological state and acts through a diversity of signaling pathways. This adhesion molecule exerts a crucial function to regulate multistep processes, including migration, proliferation, and metastasis (31, 53–55). Integrins consist of two subunits, α and β, so they are heterodimers. On cell membranes, they can interact with extracellular ligands and serve as receptors to mediate intracellular signals. As adhesion proteins, integrins are engaged in a variety of cellular functions (56). If the local responses are disturbed, integrin activation may occur, leading to tissue damage and inflammation (57). ITGA9, which is formed of α9 and β1 subunits, is one of the less known. Its role in nociceptive transmission needs to be studied, which is why we focused on this topic in our work. The results of our research revealed for the first time that after CCI, the protein level of ITGA9 remains at a similar level in the cytoplasmic fraction and is lowered in the membrane fraction, which may be caused by the internalization of the receptor after its activation by XCL1 in the course of neuropathy. Our immunofluorescence staining indicated the presence of ITGA9 in spinal neurons but not in microglial and astroglial cells. The importance of this receptor in nociceptive transmission was proven for the first time by our pharmacological research. The spinal blockade of ITGA9 by YA4 significantly diminished both thermal and mechanical hypersensitivity evoked by CCI. The results are promising because it was already shown that blocking ITGA9 has beneficial effects in mouse models of experimental autoimmune encephalomyelitis (31) and arthritis (58). According to some literature data from *in vitro* studies, XCL1 signaling *via* ITGA9 may be neuroprotective by increasing the number of neurospheres, promoting neuronal differentiation and positively affecting neurogenesis (59); it also potentiates the regeneration of axons (60). On the other hand, some researchers have suggested that its blockade can reverse the outcomes of autoimmune diseases (31). Therefore, in our opinion, ITGA9 is an interesting target for pharmacotherapy, and it is definitely tempting to look for new pharmacological tools for its modulation.

Opioids are used in chronic pain treatment, but in neuropathy, they exhibit lower effectiveness (16, 61). Many studies have proven that pharmacological inhibition of glial activation in neuropathy provides beneficial effects on opioid analgesic efficacy (4, 62). Initially, the reason for this phenomenon was the reduced release of cytokines (including chemokines) by these cells (16, 61). Recent data in the literature confirmed this theory, showing that intrathecal administration of CCL2- and CCL7 neutralizing antibodies enhanced the analgesic effects of morphine and buprenorphine in CCI-exposed mice (19). Moreover, in cancer-induced bone pain, inhibition of CXCL10, CXCL11 and CXCL13 enhanced morphine analgesic properties in rats (63–65). Moreover, in diabetic neuropathic pain, intrathecal administration of CCL1-, CCL3- and CCL9 neutralizing antibodies enhanced morphine effectiveness (18, 20). Our results provide the first evidence that an XCL1 neutralizing antibody improves morphine (a strong agonist of MOR, with lower affinity to DOR, KOR), but not buprenorphine (an agonist of MOR/NOR; antagonist of KOR/DOR), analgesia in CCI-exposed mice. Apart from the fact that these opioids acting through different receptors, which might be the explanation for why neutralization of XCL1 differently affects their analgesic properties, it is important to keep in mind that they also showed different pharmacokinetics and pharmacodynamics. Additionally it is known that chemokine receptors may create heterodimers with the opioid receptors MOR and DOR, which are involved in morphine analgesia (66–68), however, no such data are available for NOR. Some chemokines (e.g., CCL2, CCL5, CXCL12, CX3CL1) have already been known to interfere with the analgesic effects induced by morphine, DAMGO (selective ligand of MOR) and/or DPDPE (selective ligand of DOR) due to heterologous desensitization (67, 68). To date, there are no reports that have documented heterologous desensitization of NOR and chemokine receptors, but this mechanism could be one of the reasons why buprenorphine analgesia is improved by the blockade of XCR1. However, this topic requires further in-depth investigation. We previously proved that the blockade of other typical chemokine receptors, such as CCR1 by J113863 (21), CCR2 by RS504393 (22) and CCR5 by maraviroc (69), enhanced the analgesic properties of morphine and buprenorphine under neuropathy. What may be surprising is that a blockade of ITGA9 acts similarly, which enhances the analgesic effects of morphine and – to a greater extent – the latter buprenorphine. However, this is not a typical chemokine receptor. It was already shown that some integrins play an important role in the modulation of opioid signaling in trigeminal ganglion neurons. The β1 integrin subunit (present in ITGA9) was shown to have a high degree of colocalization with MOR in these neurons (70). These are particularly significant results since they indicate for the first time that ITGA9 may be an important potential target for the pharmacotherapy of neuropathy. Therefore, our results are particularly important because ITGA9 is not a typical chemokine receptor, which indicates the complexity of neuroimmunological processes occurring in neuropathy. We assume that one of the mechanisms underlying the beneficial properties of chemokine receptor blockers/antagonists is to prevent the anti-opioid effects of chemokines. The inhibition of neuroimmune imbalance may contribute to a potential therapeutic mechanism based on increasing the efficacy of opioids in neuropathic pain treatment.

## 5 Conclusion

Based on the current results, we can confirm that XCL1/XCR1 and XCL1/ITGA9 signaling play important roles in CCI-induced neuropathy; however, ITGA9 seems to be a more potent neuronal target and may serve as an innovative strategy for the polypharmacotherapy of neuropathic pain in combination with opioids. Moreover, our data suggest that minocycline, a widely used antibiotic that affects many intracellular pathways, can reveal high analgesic potential in neuropathy by influencing more immune factors than was previously thought, including XCL1. In view of the obtained data and current literature, we suggest that modulation of XCL1 signaling may serve as a promising target for combined therapy with opioids and indicates minocycline repurposing potential in the treatment of neuropathic pain. Moreover, both XCL1 receptors (XCR1 and ITGA9), seem to be important novel targets with beneficial properties for pharmacological intervention after nerve injury. Both used pharmacological tools (MIP-II protein, YA4) are available for experimental studies, but as far as we know, they are not drugs used in the clinics. That is why we need more studies, taking into consideration neuropathic pain of different etiologies, especially in the light of the knowledge that both, chemokines neutralization and neutralization/blockade of their receptors are successfully used as a treatment of varied diseases.

## Data availability statement

The original contributions presented in the study are included in the article/**Supplementary Material**. Further inquiries can be directed to the corresponding author.

## Ethics statement

The animal study was reviewed and approved by Ethical Committee of the Maj Institute of Pharmacology of the Polish Academy of Sciences (LKE: 75/2017, 305/2017, 235/2020, 236/2021, 297/2021, 89/2021, 98/2022). According to the 3R policy, the number of animals was reduced to the necessary minimum.

## Author contributions

AC, ER, AP, JB, KP, KC, GK and JM substantially contributed to the conception and design of the study and to the analysis and interpretation of the data. All authors contributed to the article and approved the submitted version.
