# Supplementary material for: New insights into the analgesic properties of the XCL1/XCR1 and XCL1/ITGA9 axes modulation under neuropathic pain conditions - evidence from animal studies
Source: Front Immunol. 2022 Dec 22;13:1058204. doi: 10.3389/fimmu.2022.1058204 (PMC9814969; doi:10.3389/fimmu.2022.1058204)
Supplement: Supplementary file 1 [file DataSheet_1.pdf]

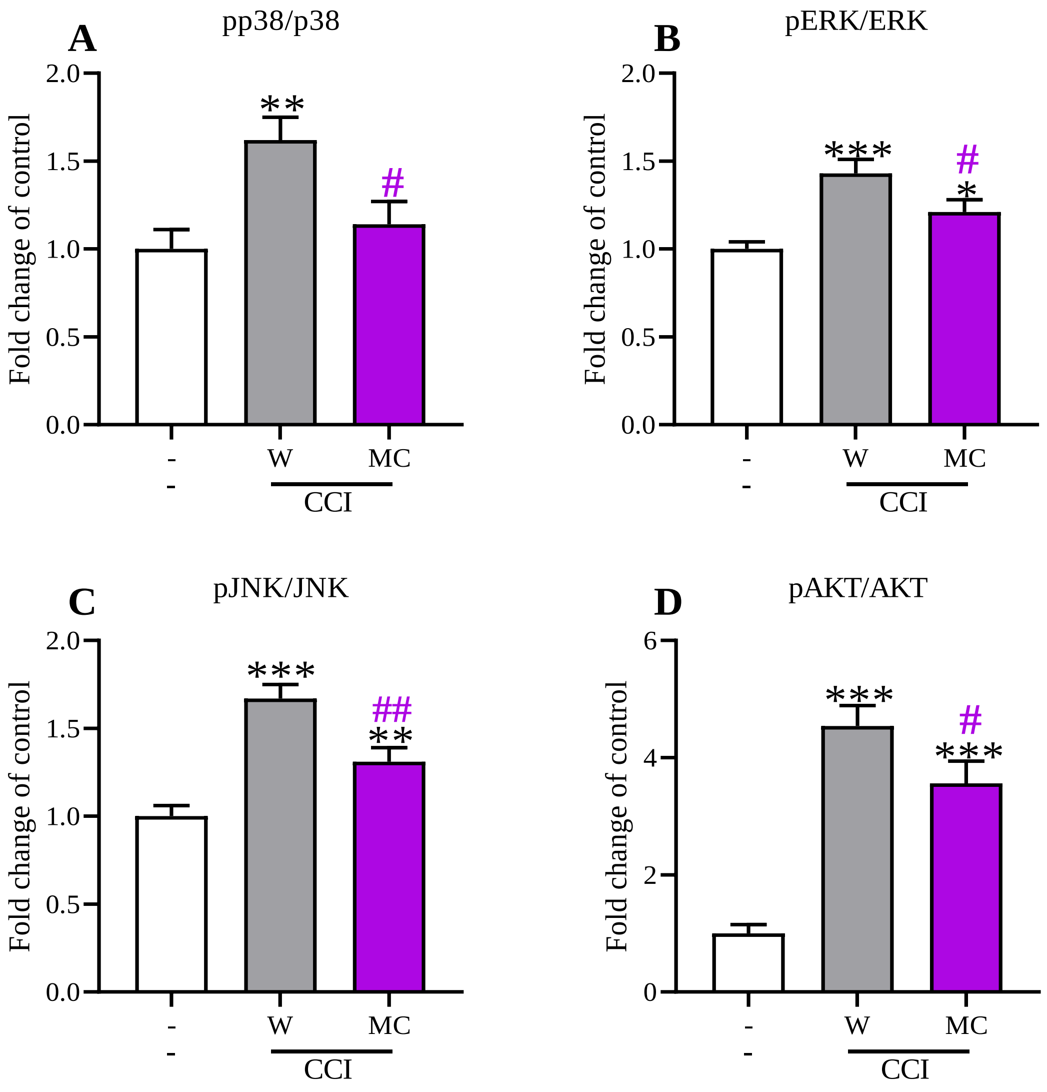

Fig 1. The influence of repeated (preemptive and then twice daily for 7 days) minocycline (MC) administrations, at a dose 30 mg/kg, on pp38/p38 (**A**), pERK/ERK (**B**); pJNK/JNK (**C**), pAKT/AKT (**D**) protein level in cytoplasmic fraction by Luminex, 7 days after chronic constriction injury of the sciatic nerve in mice. The data are presented as mean fold changes relative to the control ± SEM (n=6–10). The results were evaluated using one-way Anova followed by Bonferroni's *post hoc* test for comparisons of selected pairs; *$p < 0.05$; **$p < 0.01$; ***$p < 0.001$ indicate significant differences between the naive vs. W–/MC–treated groups; #$p < 0.05$; ##$p < 0.01$ indicate significant differences between the W– vs. MC–treated groups. Abbreviations: "-"– naive; "W" – vehicle (water for injections).

Naive, ipsilateral

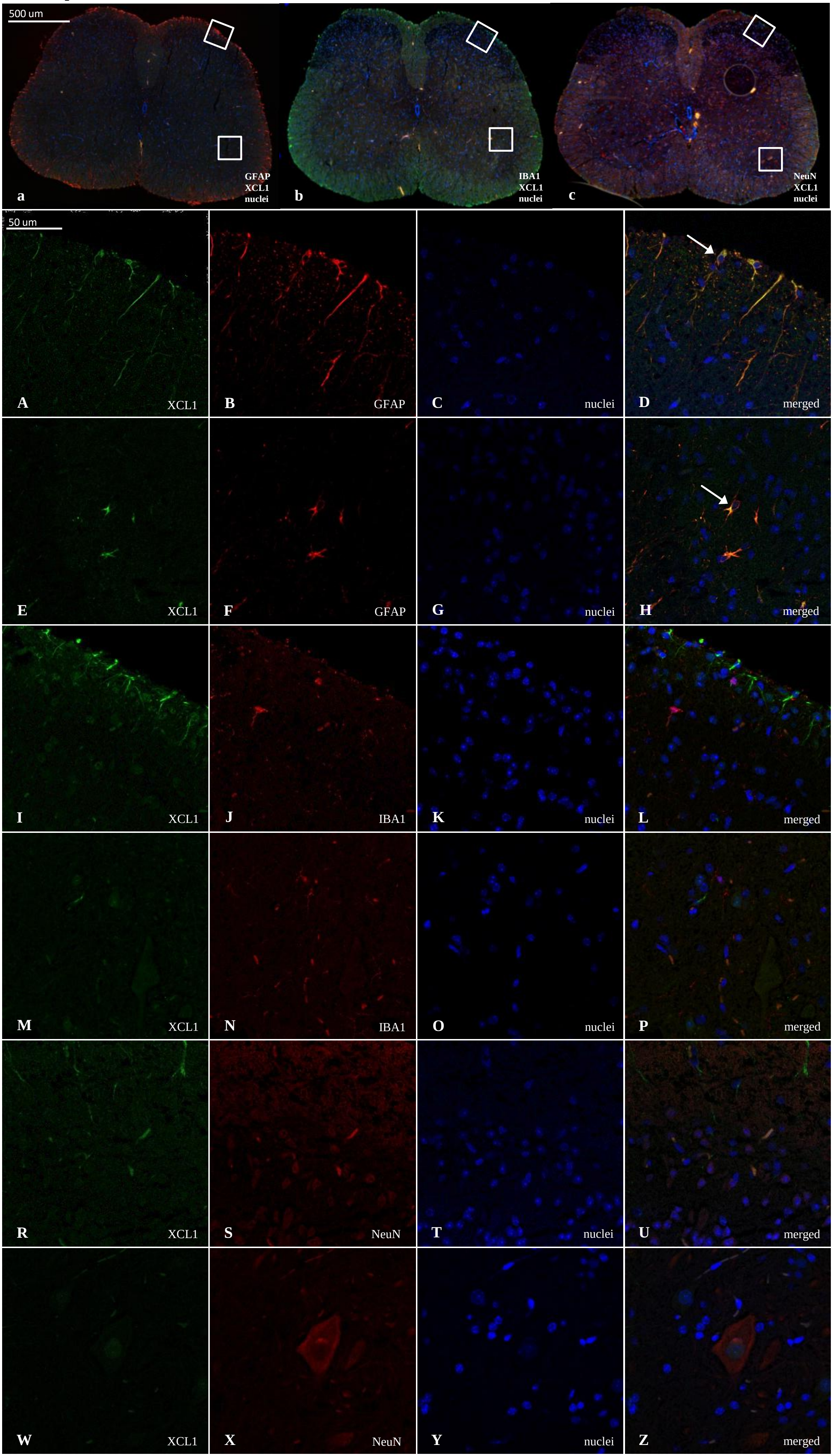

Fig 2. Immunohistochemical analysis of chemokine-C-motif ligand 1 (XCL1) localization in the lumbar (L4 to L6) spinal cord of naive mice. Dorsal (**A,B,C,D,I,J,K,L,R,S,T,U**) and ventral (**E,F,G,H,M,N,O,P,W,X,Y,Z**) parts of lumbar spinal cord were shown as an approximate fragments of selected images. Representative immunofluorescent images from colocalization analysis performed on spinal cord, paraffin-embedded 7 µM microtome slices: XCL1 (green: **A,E,I,M,R,W**) with astroglia marker glial fibrillary acidic protein; (GFAP, red: **B,F**); microglia marker ionized calcium-binding adaptor molecule 1; (IBA1, red: **J,N**); and with neuronal marker neuronal nucleus; (NeuN, red: **S,X**), Nuclei are in blue (**C,G,K,O,T,Y**). Scale bars: 50 µm (**a, b, c**), 500 µm (**A-Z**).

Naive, ipsilateral

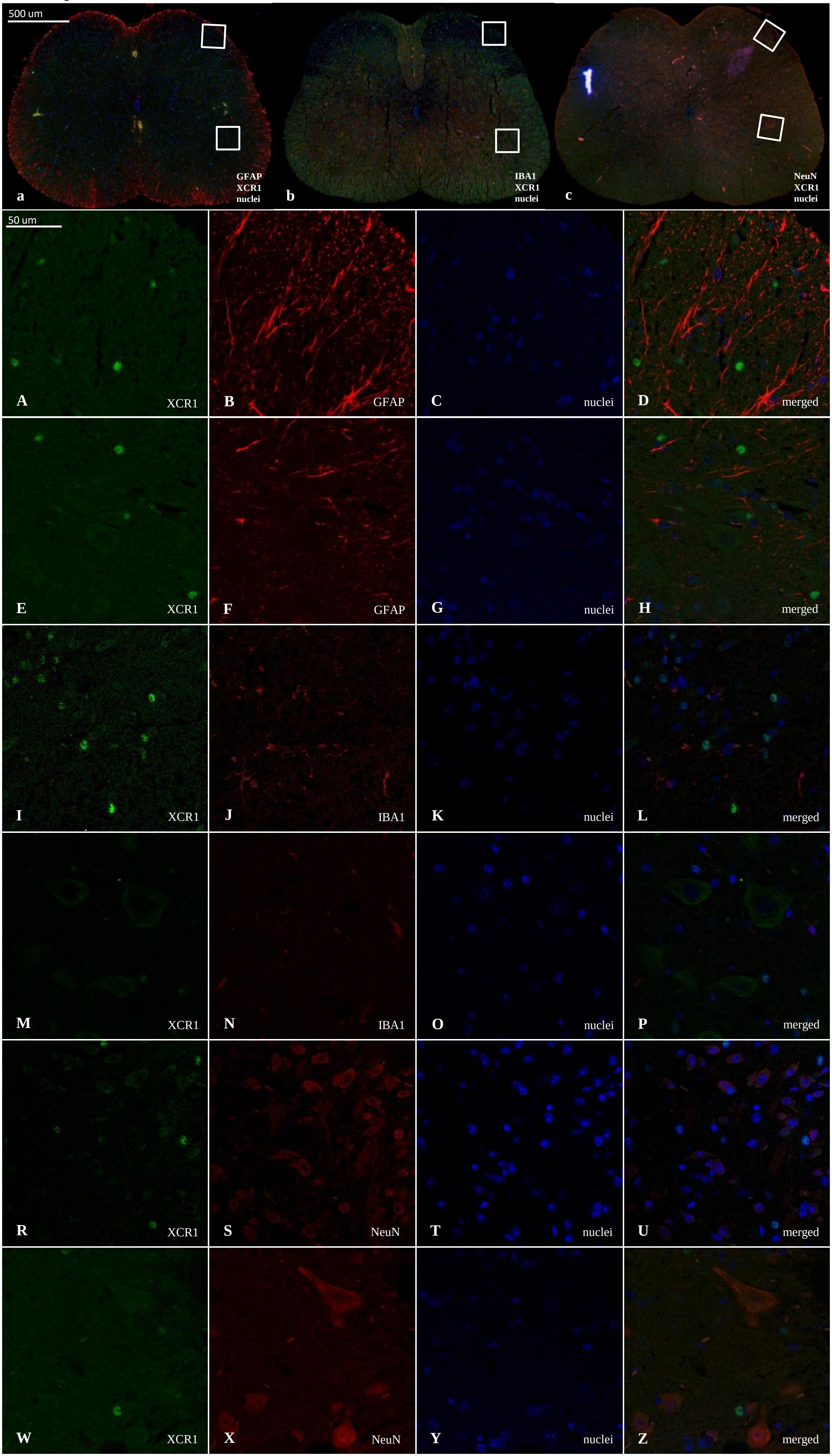

Fig 3. Immunohistochemical analysis of chemokine-C-motif receptor 1 (XCR1) localization in the lumbar (L4 to L6) spinal cord of naive mice. Dorsal (**A,B,C,D,I,J,K,L,R ,S,T,U**) and ventral (**E,F,G,H,M,N,O,P,W,X,Y,Z**) parts of lumbar spinal cord were shown as an approximate fragments of selected images. Representative immunofluorescent images from colocalization analysis performed on spinal cord, paraffin-embedded 7 μM microtome slices: XCR1 (green: **A,E,I,M,R,W**) with astroglia marker glial fibrillary acidic protein; (GFAP, red: **B,F**); microglia marker ionized calcium-binding adaptor molecule 1; (IBA1, red: **J,N**); and with neuronal marker neuronal nucleus; (NeuN, red: **S,X**), Nuclei are in blue (**C,G,K,O,T,Y**). Scale bars: 50 μm (**a, b, c**), 500 μm (**A-Z**).

Naive, ipsilateral

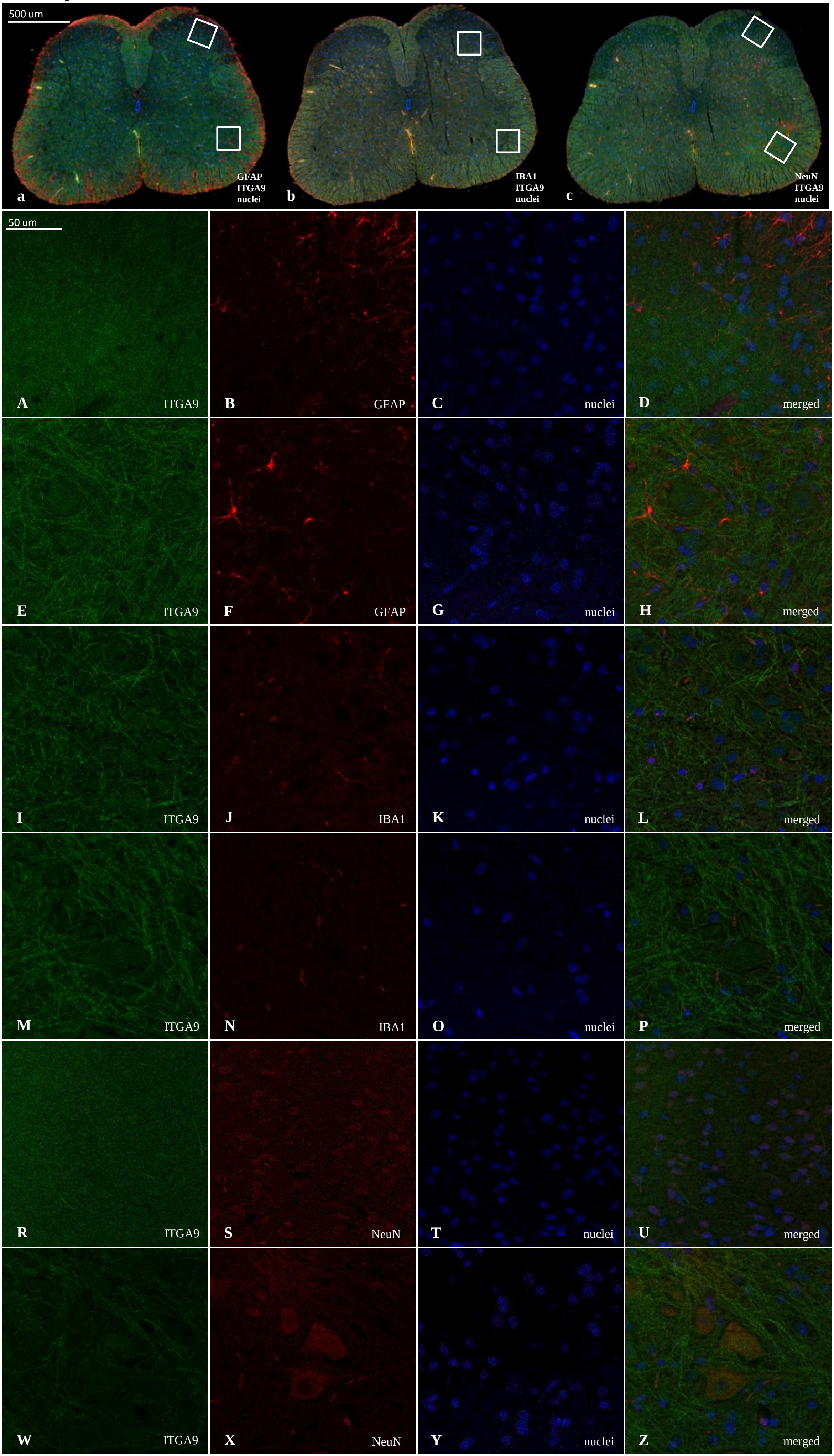

Fig 4. Immunohistochemical analysis of integrin alpha-9 (ITGA9) localization in the lumbar (L4 to L6) spinal cord of naive mice. Dorsal (**A,B,C,D,I,J,K,L,R,S,T,U**) and ventral (**E,F,G,H,M,N,O,P,W,X,Y,Z**) parts of lumbar spinal cord were shown as an approximate fragments of selected images. Representative immunofluorescent images from colocalization analysis performed on spinal cord, paraffin-embedded 7 μM microtome slices: ITGA9 (green: **A,E,I,M,R,W**) with astroglia marker glial fibrillary acidic protein; (GFAP, red: **B,F**); microglia marker ionized calcium-binding adaptor molecule 1; (IBA1, red: **J,N**); and with neuronal marker neuronal nucleus; (NeuN, red: **S,X**), Nuclei are in blue (**C,G,K,O,T,Y**). Scale bars: 50 μm (**a, b, c**), 500 μm (**A-Z**).
