# Supplementary material for: New insights into the analgesic properties of the XCL1/XCR1 and XCL1/ITGA9 axes modulation under neuropathic pain conditions - evidence from animal studies
Source: Front Immunol. 2022 Dec 22;13:1058204. doi: 10.3389/fimmu.2022.1058204 (PMC9814969; doi:10.3389/fimmu.2022.1058204)
Supplement: Supplementary file 2 [file DataSheet_2.pdf]

protein homogenate

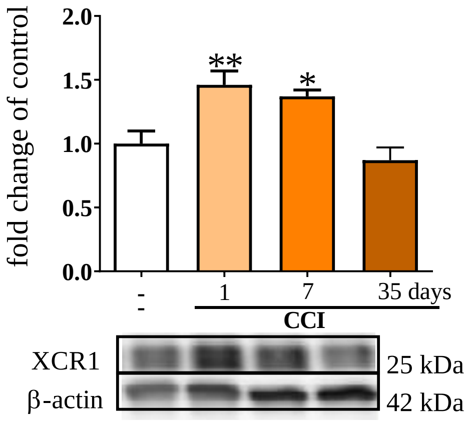

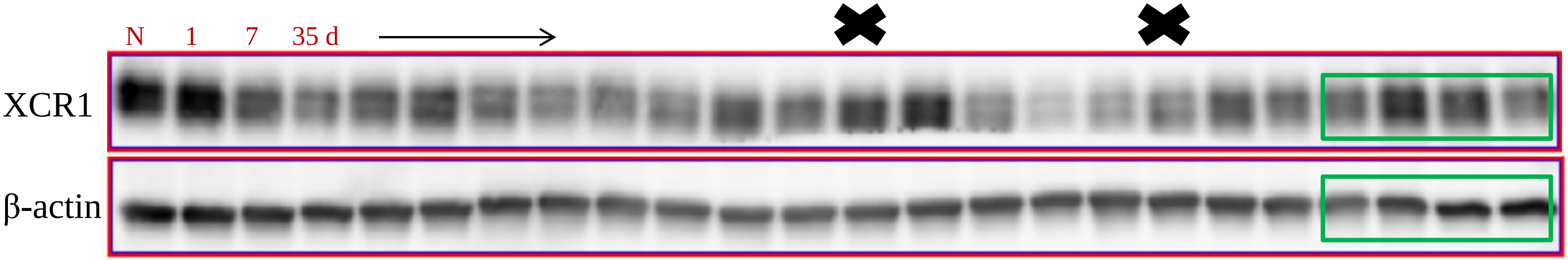

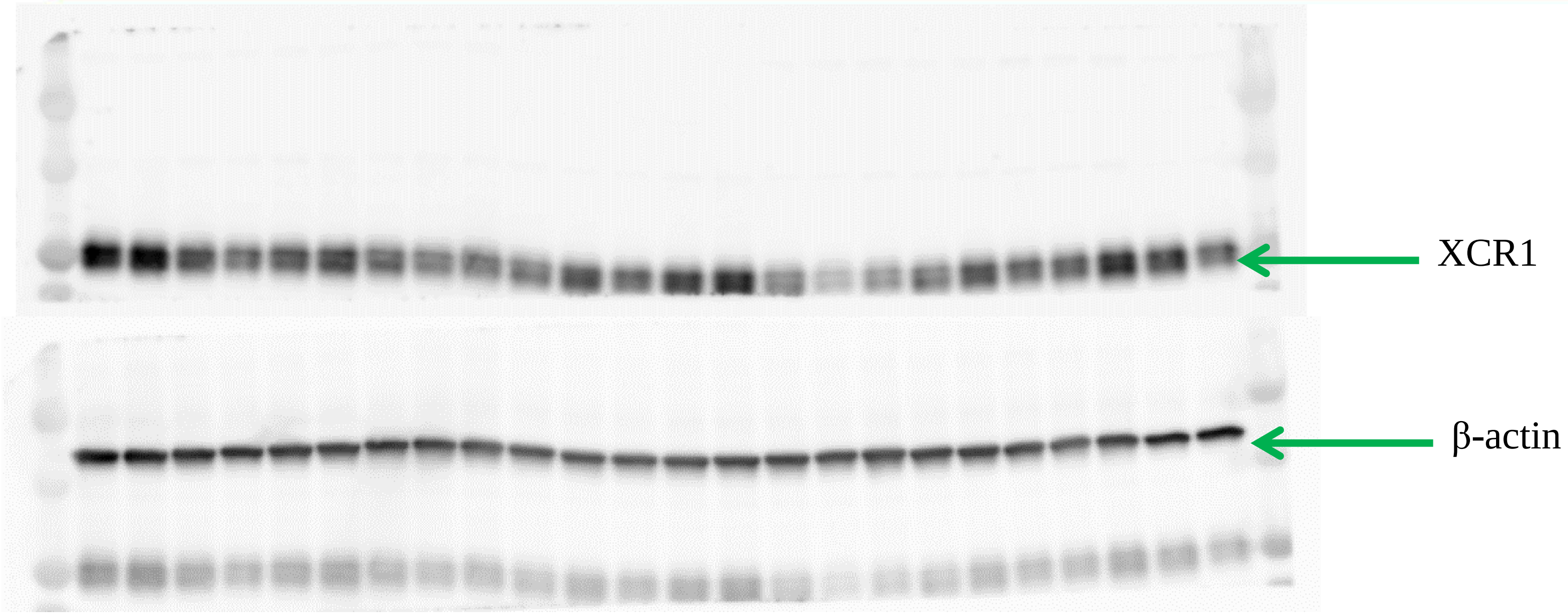

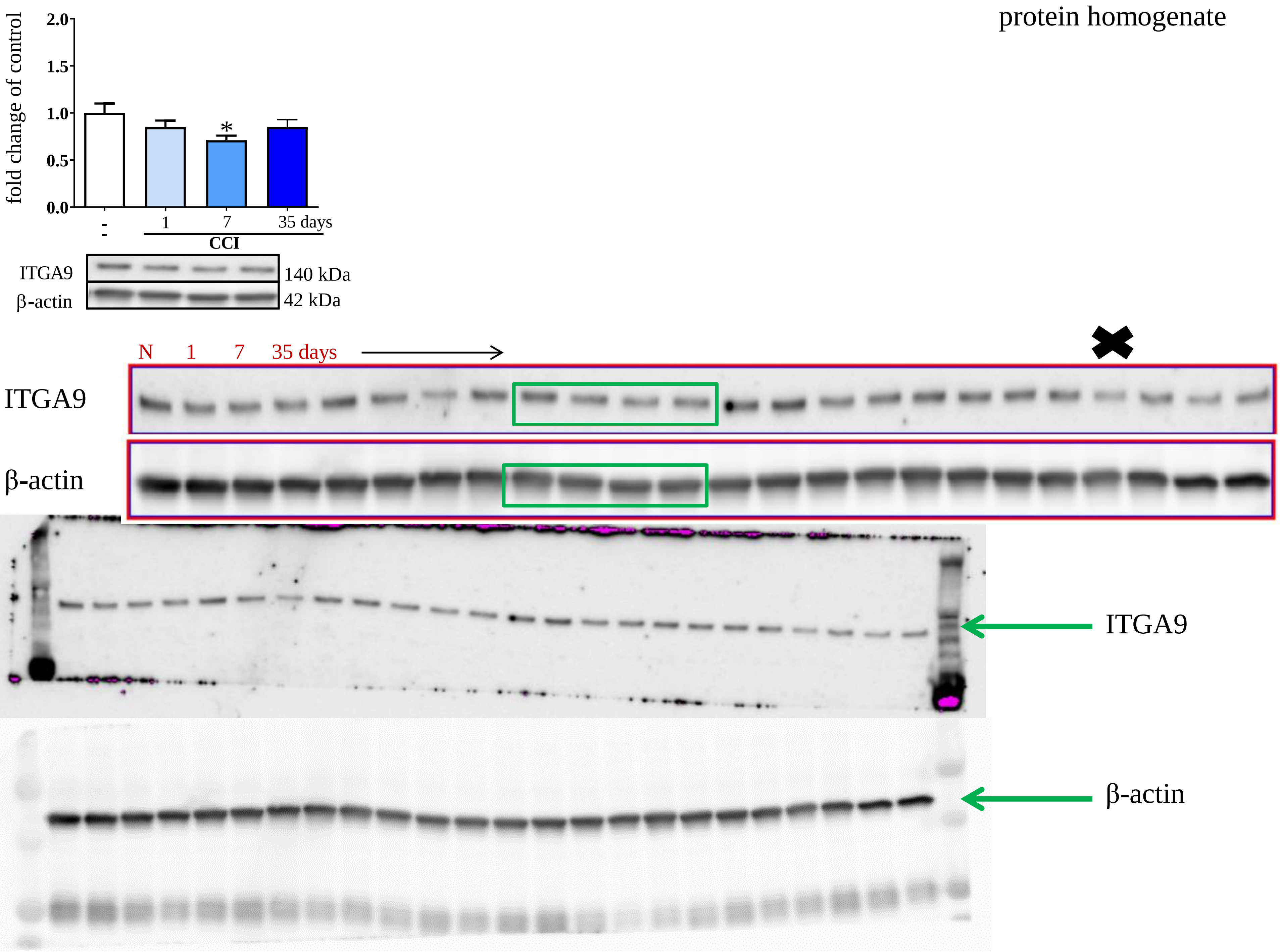
protein homogenate
fold change of control
2.0
1.5
1.0
0.5
0.0
*
-
1
7
35 days
CCI
ITGA9
β-actin
140 kDa
42 kDa
N
1
7
35 days
ITGA9
β-actin
ITGA9
β-actin

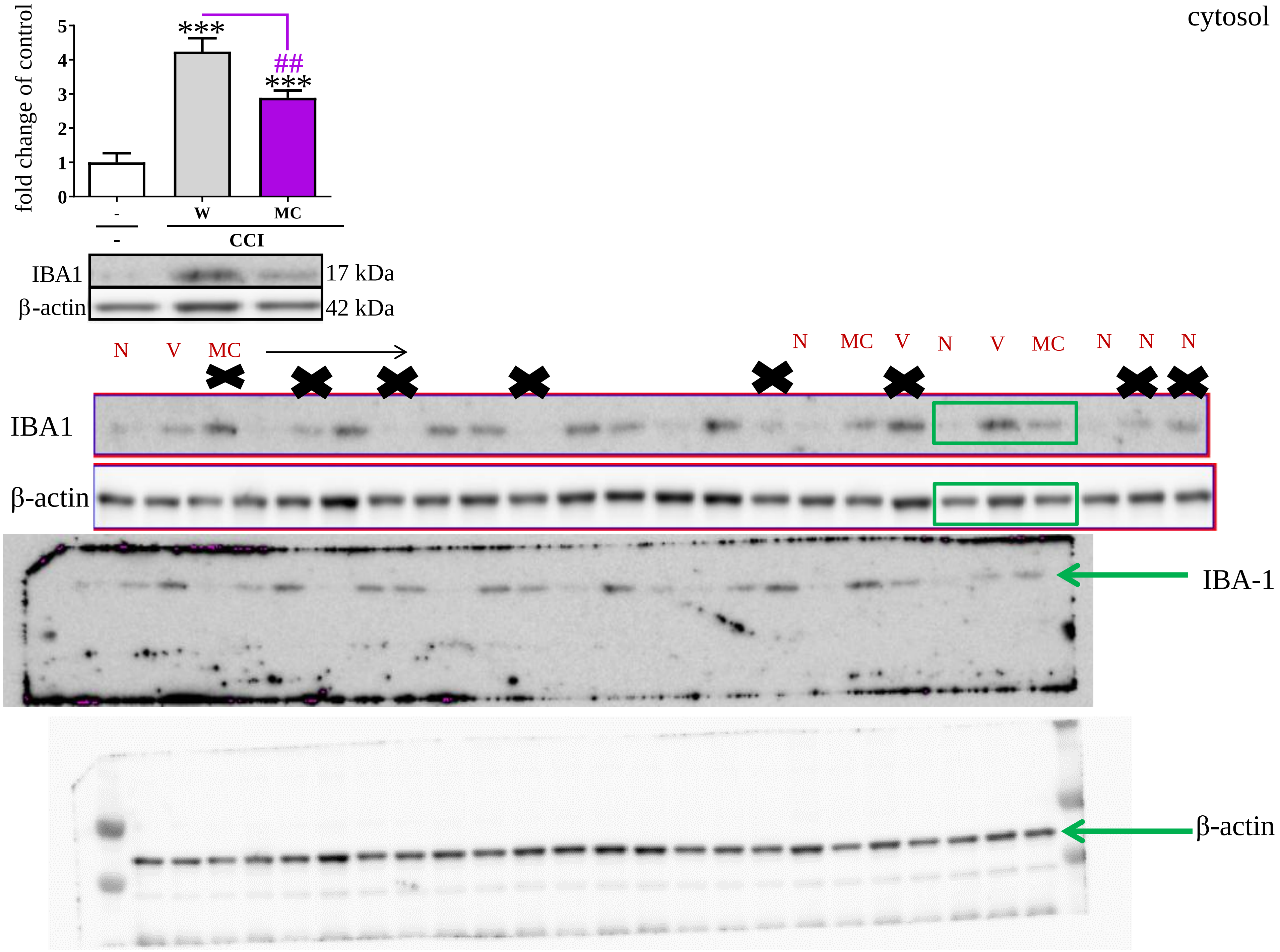
cytosol
fold change of control
0
1
2
3
4
5
***
##
***
-
W
MC
-
CCI
IBA1
β-actin
17 kDa
42 kDa
N
V
MC
N
MC
V
N
V
MC
N
N
N
IBA1
β-actin
IBA-1
β-actin

cytosol

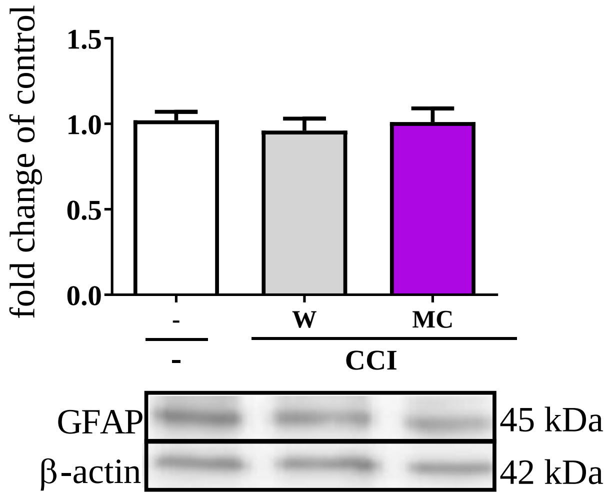
fold change of control
1.5
1.0
0.5
0.0
-
W
MC
-
CCI
GFAP
β-actin
45 kDa
42 kDa

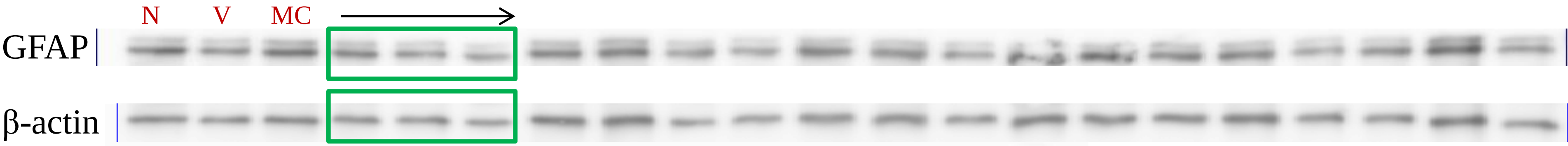
N
V
MC
GFAP
β-actin

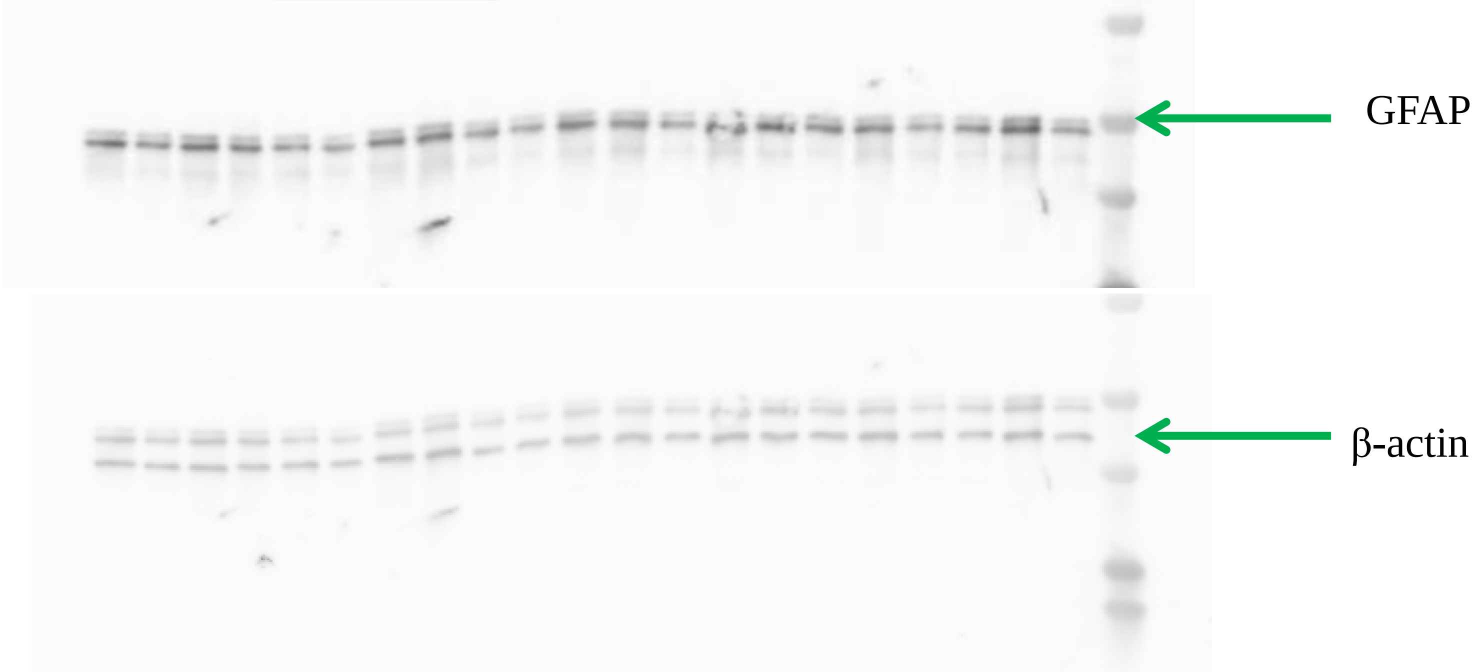
GFAP
β-actin

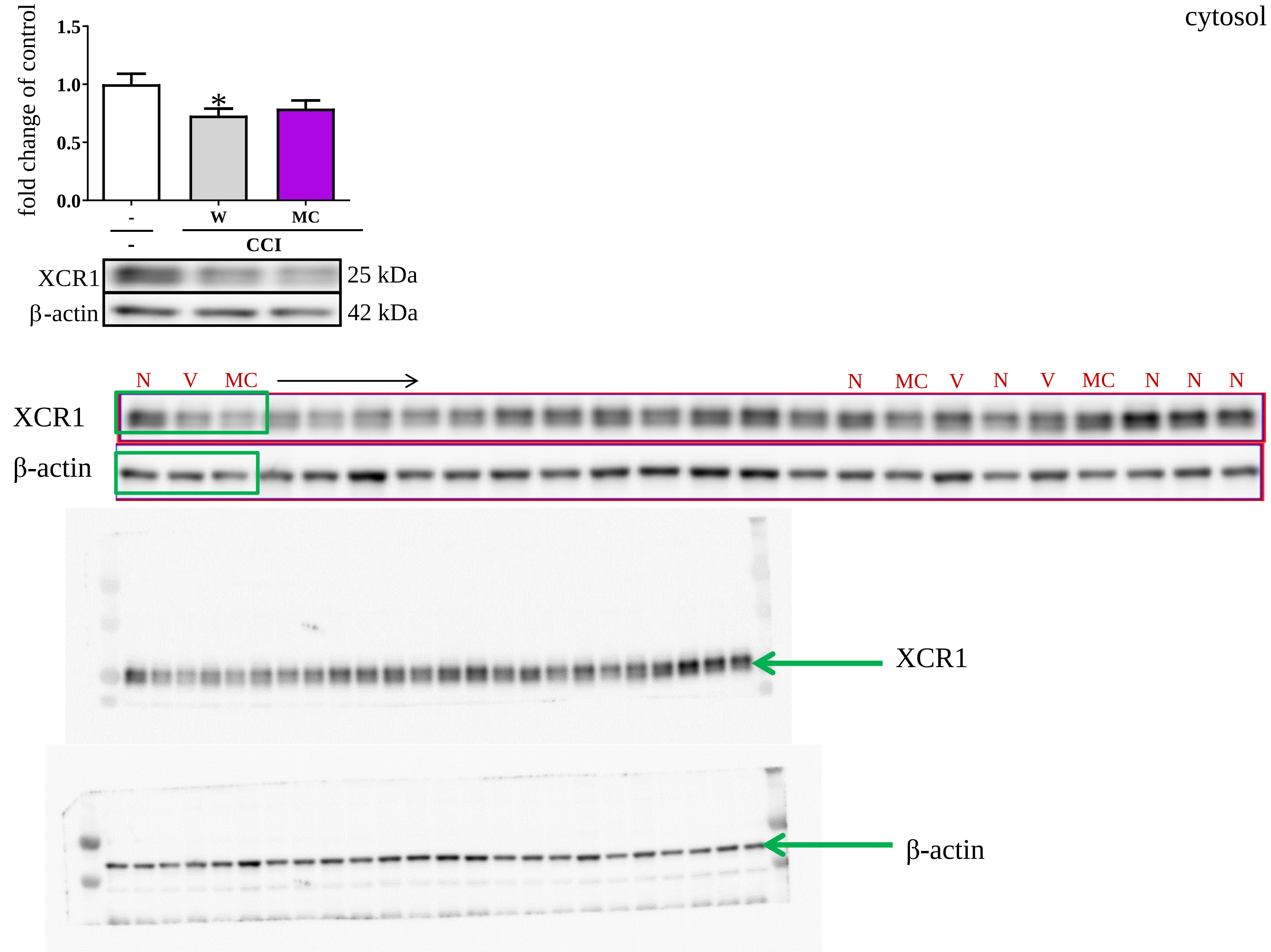
cytosol
fold change of control
1.5
1.0
0.5
0.0
*
-
W
MC
-
CCI
XCR1
β-actin
25 kDa
42 kDa
N
V
MC
N
MC
V
N
V
MC
N
N
N
XCR1
β-actin
XCR1
β-actin

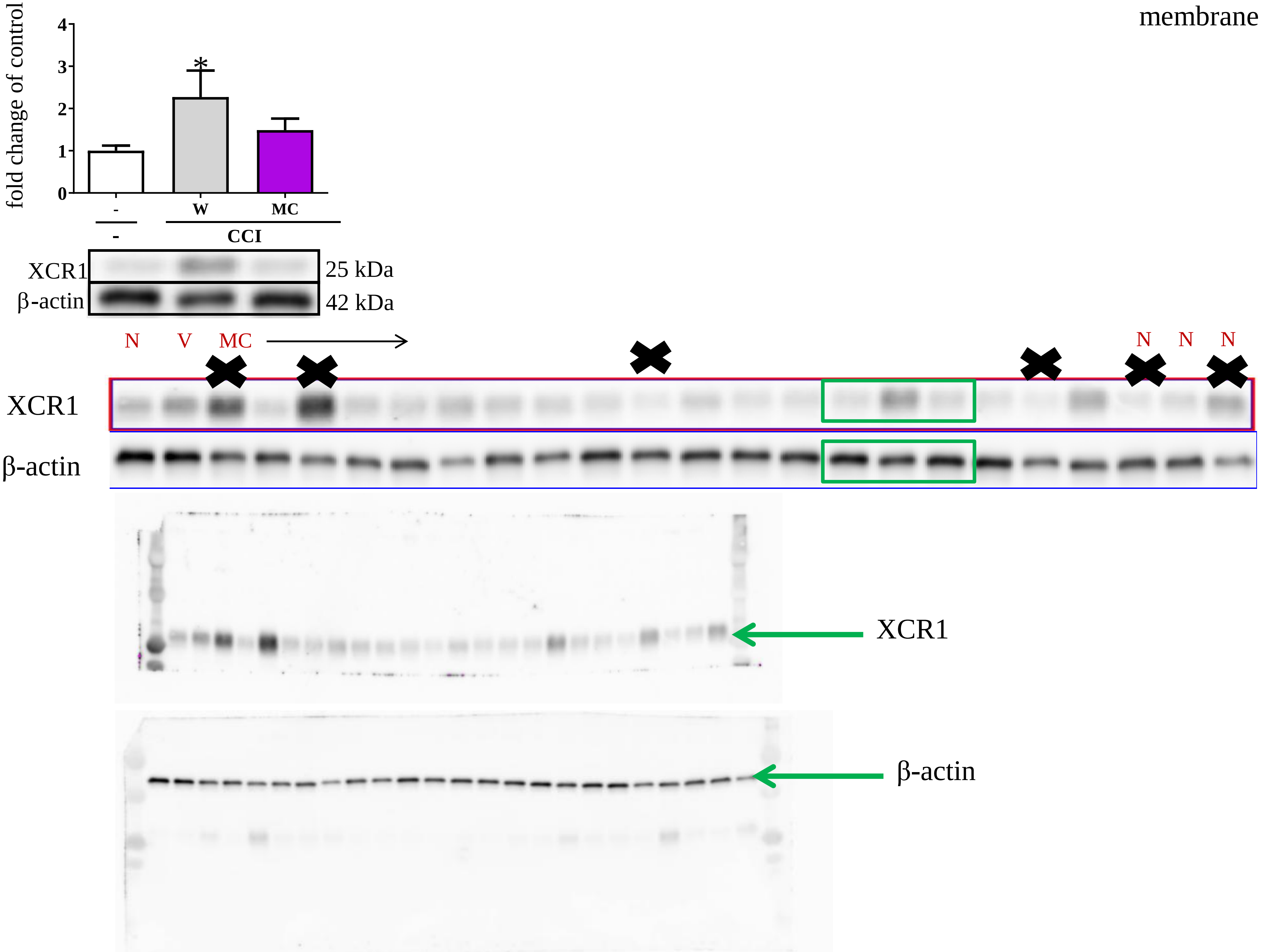

membrane
fold change of control
4
3
2
1
0
*
-
W
MC
-
CCI
XCR1
β-actin
25 kDa
42 kDa
N
V
MC
N
N
N
XCR1
β-actin
XCR1
β-actin

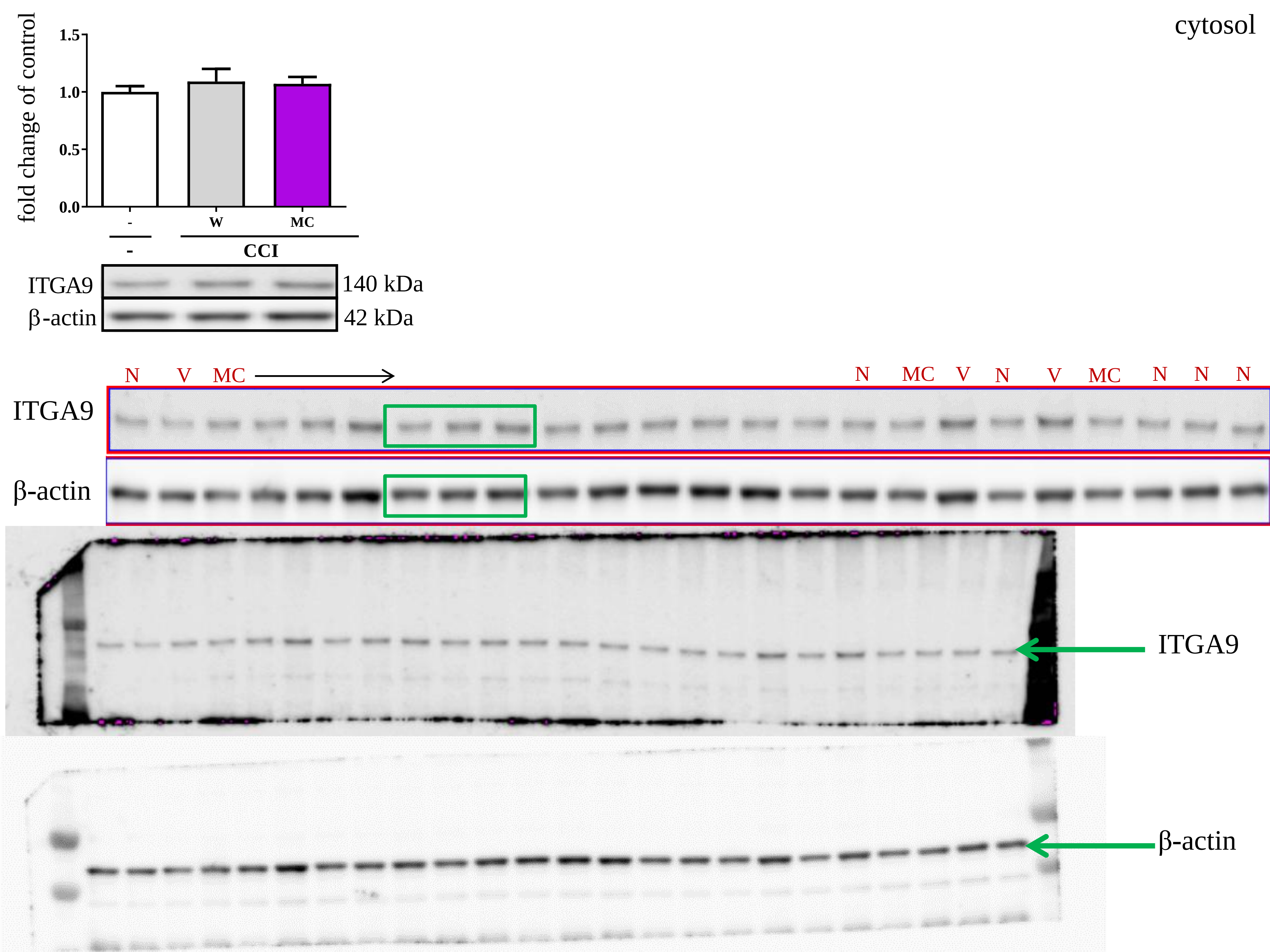

cytosol
fold change of control
1.5
1.0
0.5
0.0
-
W
MC
-
CCI
ITGA9
β-actin
140 kDa
42 kDa
N
V
MC
N
MC
V
N
V
MC
N
N
N
ITGA9
β-actin
ITGA9
β-actin

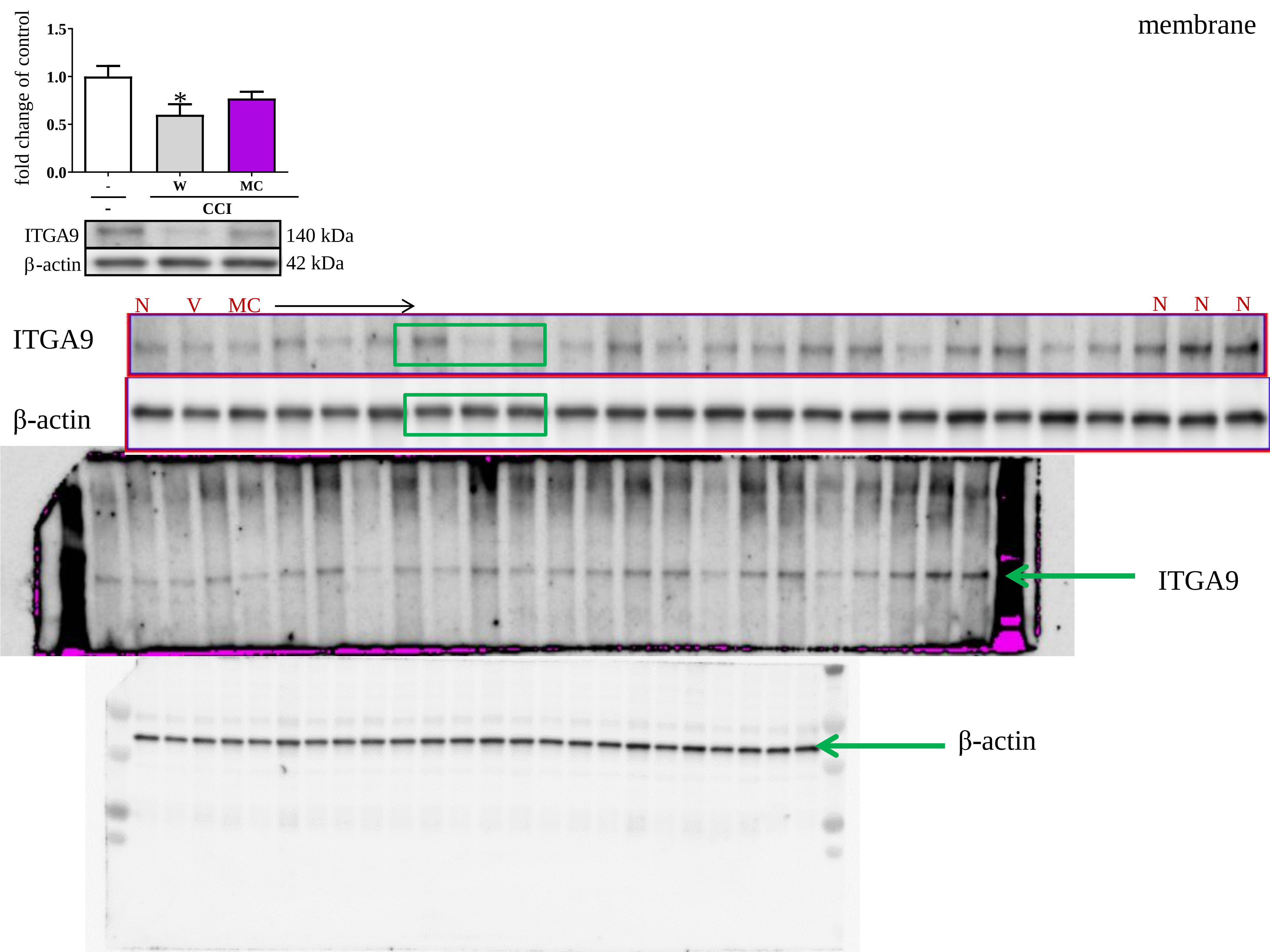

membrane
fold change of control
1.5
1.0
0.5
0.0
*
-
W
MC
-
CCI
ITGA9
β-actin
140 kDa
42 kDa
N
V
MC
N
N
N
ITGA9
β-actin
ITGA9
β-actin
